# Pilus Production in Acinetobacter baumannii Is Growth Phase Dependent and Essential for Natural Transformation

**DOI:** 10.1128/JB.00034-21

**Published:** 2021-03-23

**Authors:** Nina Vesel, Melanie Blokesch

**Affiliations:** a Laboratory of Molecular Microbiology, Global Health Institute, School of Life Sciences, Ecole Polytechnique Fédérale de Lausanne (EPFL), Lausanne, Switzerland; Princeton University

**Keywords:** *Acinetobacter baumannii*, natural competence for transformation, twitching motility, type IV pili

## Abstract

Acinetobacter baumannii is a severe threat to human health as a frequently multidrug-resistant hospital-acquired pathogen. Part of the danger from this bacterium comes from its genome plasticity and ability to evolve quickly by taking up and recombining external DNA into its own genome in a process called natural competence for transformation. This mode of horizontal gene transfer is one of the major ways that bacteria can acquire new antimicrobial resistances and toxic traits. Because these processes in A. baumannii are not well studied, we herein characterized new aspects of natural transformability in this species that include the species’ competence window. We uncovered a strong correlation with a growth phase-dependent synthesis of a type IV pilus (TFP), which constitutes the central part of competence-induced DNA uptake machinery. We used bacterial genetics and microscopy to demonstrate that the TFP is essential for the natural transformability and surface motility of A. baumannii, whereas pilus-unrelated proteins of the DNA uptake complex do not affect the motility phenotype. Furthermore, TFP biogenesis and assembly is subject to input from two regulatory systems that are homologous to Pseudomonas aeruginosa, namely, the PilSR two-component system and the Pil-Chp chemosensory system. We demonstrated that these systems affect not only the piliation status of cells but also their ability to take up DNA for transformation. Importantly, we report on discrepancies between TFP biogenesis and natural transformability within the same genus by comparing data for our work on A. baumannii to data reported for Acinetobacter baylyi, the latter of which served for decades as a model for natural competence.

**IMPORTANCE** Rapid bacterial evolution has alarming negative impacts on animal and human health which can occur when pathogens acquire antimicrobial resistance traits. As a major cause of antibiotic-resistant opportunistic infections, A. baumannii is a high-priority health threat which has motivated renewed interest in studying how this pathogen acquires new, dangerous traits. In this study, we deciphered a specific time window in which these bacteria can acquire new DNA and correlated that with its ability to produce the external appendages that contribute to the DNA acquisition process. These cell appendages function doubly for motility on surfaces and for DNA uptake. Collectively, we showed that A. baumannii is similar in its TFP production to Pseudomonas aeruginosa, though it differs from the well-studied species A. baylyi.

## Abstract (precis)

Rapid bacterial evolution has alarming negative impacts on animal and human health which can occur when pathogens acquire antimicrobial resistance traits. As a major cause of antibiotic-resistant opportunistic infections, A. baumannii is a high-priority health threat which has motivated renewed interest in studying how this pathogen acquires new, dangerous traits.

## INTRODUCTION

Bacterial evolution is a major human health concern, as it can lead to the acquisition of concerning traits, such as new antimicrobial resistances or virulence genes. One pathogen of concern is the hospital-prevalent antimicrobial-resistant Acinetobacter baumannii (1, 2), which evolves rapidly by incorporating significant amounts of DNA from other organisms in a process called horizontal gene transfer (HGT) (3). Using a type of HGT called natural competence for transformation, A. baumannii is able to take up extracellular DNA from its environment and incorporate it into its own genome by homologous recombination (4–7). We recently reported that such transformation events can lead to frequent exchanges of genomic regions greater than 100 kbp in the naturally competent bacterium Vibrio cholerae, which could explain how bacteria such as A. baumannii acquire new DNA stretches including resistances. However, few reports have addressed natural competence directly in A. baumannii, instead extrapolating its behavior based off that of the soil bacterium Acinetobacter baylyi (8, 9).

The few studies on transformation in A. baumannii have focused mainly on mild competence inducers such as serum albumin and Ca^2+^, on transforming materials, and on pH (10–12). However, transforming protocols vary wildly between studies (13–15), including the use of different solidifying agents for transformation scoring on surfaces (16). Additionally, only few isolates of this species, such as the strains A118 (13) and M2 (recently reclassified as Acinetobacter nosocomialis) (15, 17), were previously found to be naturally competent, though recent studies are showing that a plethora of clinical and wildlife/livestock A. baumannii isolates are likewise naturally transformable (12, 14, 18, 19). Therefore, the process of natural competence in A. baumannii needs to be better studied and recorded.

**FIG 1:**
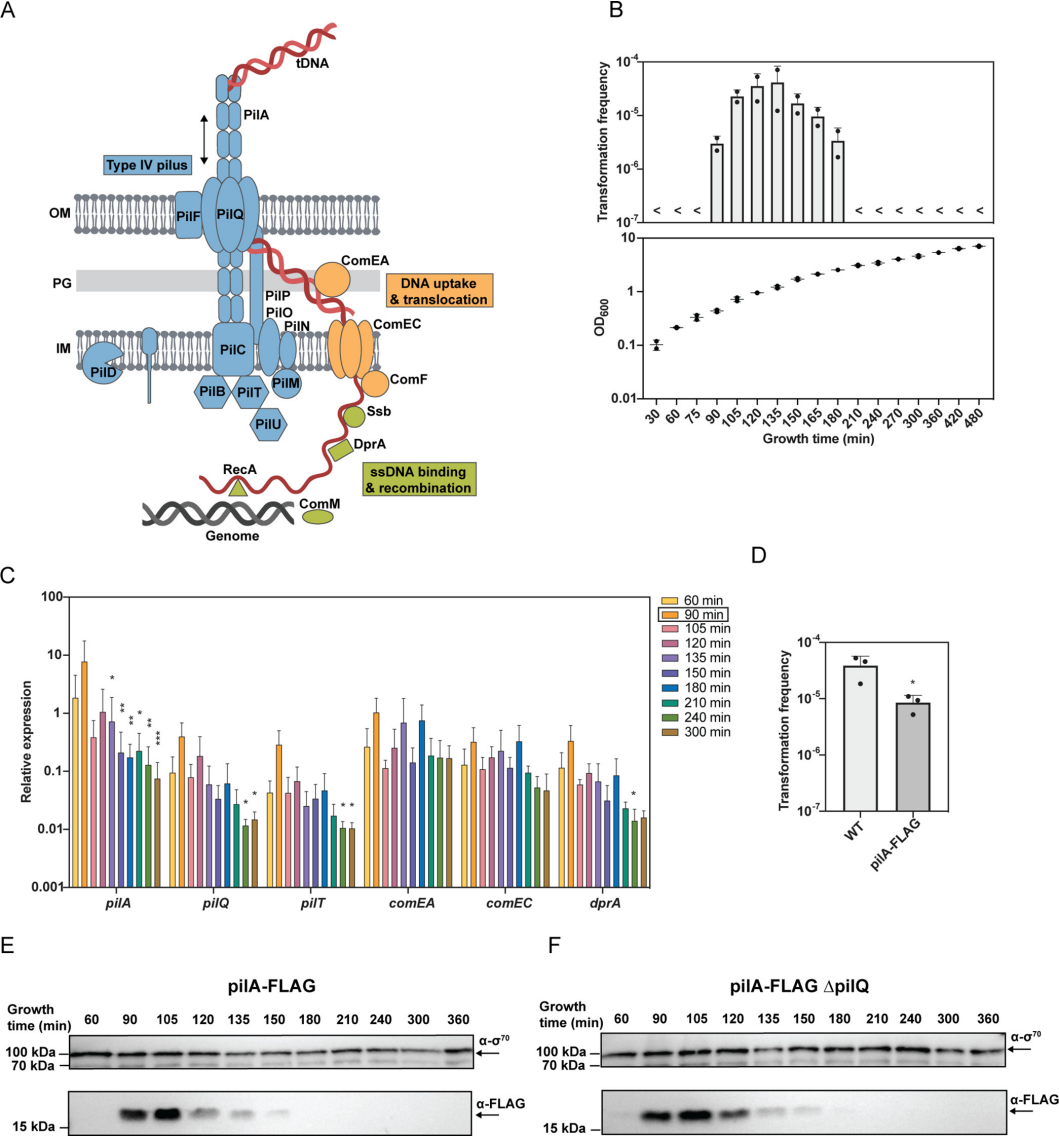
Transformation is growth phase-dependent in A. baumannii. (A) Schematic representation of DNA uptake machinery. Type IV pilus (TFP) components are shown in blue, DNA uptake and translocation proteins are in orange, and proteins for binding and recombination of the incoming ssDNA are in green. (B) Natural transformability of A. baumannii over time. The graph on top shows the transformation frequencies, while the graph on the bottom depicts the growth of the bacteria given in optical density at 600 nm (OD_600_) units. (C) Relative expression values over time for a subset of competence genes (*pilA*, *pilQ*, *pilT*, *comEA*, *comEC*, and *dprA*). (D) Transformation frequencies of the wild type (WT) and the strain encoding the PilA-FLAG translational fusion. (E and F) Immunoblotting of FLAG-tagged PilA at different time points through the bacterial growth phases (as indicated in minutes) in the *pilA*-FLAG strain (D) or its *pilQ*-negative derivative (*pilA*-FLAG Δ*pilQ*) (E). Detection of Sigma70 served as loading control. Two and three biologically independent experiments were performed for panel B and panels C to F, respectively, and the mean values (±standard deviation [SD]) are shown for all graphs. <, below detection limit. Statistical analyses were performed on log-transformed data. Statistics were based on a two-way analysis of variance (ANOVA) test with Tukey’s multiple comparisons (time points compared to 90 min, as indicated by the box in the legend) (C) or an unpaired *t* test with Welch’s correction (D). *, *P* < 0.05; **, *P* < 0.01; ***, *P* < 0.001.

Additionally, a number of studies have shown the induction of transformation potential in a surface-dependent manner, suggesting a correlation between natural transformability and the movement of A. baumannii on (wet) surfaces (14, 15). This correlation is thought to be based on the bacterium’s ability to produce type IV pili (TFP) (15), which are cell appendages that frequently constitute the central part of the DNA-uptake machinery (7) (Fig. 1A). Known to play a main role in the DNA uptake complex, the regulation of TFP production is often linked to the bacterium’s competence program (6). For instance, in V. cholerae, the TFP, which enhances the bacterium’s natural transformability, is produced only when the bacterium grows on chitinous surfaces (20–24). Considering that TFP also mediate other functions that include adhesion, motility on surfaces (i.e., twitching motility), and surface sensing (25), it makes sense that the TFP would be the link between transformability and mobility in A. baumannii.

To better understand the dual roles of this dynamic TFP, it is important to understand when it is produced in the bacterium. The TFP is composed of major (PilA) and minor pilin subunits (different kinds in diverse bacteria), and extension and retraction events are energized by the cytosolic PilB and PilT/PilU ATPases, respectively. Upon or after TFP retraction, the incoming transforming DNA enters the periplasmic space through the PilQ secretin where it is bound by the DNA-binding protein ComEA. This protein is thought to act as a Brownian ratchet, which leads to the accumulation of long stretches of DNA within the bacterium’s periplasm (22, 26). After degradation of one strand, which, in Gram-negative bacteria, is attributed to an unidentified nuclease or a periplasmic extension of the ComEC protein, the single-stranded DNA translocates across the inner membrane through the ComEC channel aided by the ComF protein (Fig. 1A). Once in the cytosol, the single-stranded DNA is bound by the single-stranded binding protein Ssb, the RecA-loading protein DprA, and RecA. RecA ultimately fosters recombination if the transforming DNA is homologous with the chromosomal DNA of the cell. ComM (a RadA homolog) assists in this process, especially if heterologous DNA is present between the homologous flanks (7, 27, 28).

There are different types of TFP regulatory systems which exist in both competent and noncompetent bacteria. A major TFP regulatory system that has been studied in Pseudomonas aeruginosa (a bacterium that was only recently reclassified as naturally transformable [29]) is the two-component system (TCS) PilSR (30–32). The current model of this TCS suggests that extended pili are sensed due to the lack of inner membrane-associated PilA subunits that would otherwise interact with the atypical sensor histidine kinase PilS. The lack of interaction stimulates PilS’s kinase activity, which leads to activation of the response regulator PilR and, ultimately, *pilA* transcription (33). This PilS-mediated pilin inventory and its impact on PilR activity also influence other coregulated processes involved in virulence and surface-associated bacterial behavior (34). The Pil-Chp chemosensory system also plays a role in TFP assembly and twitching motility (35, 36). This system is composed of 10 or more components, which are mostly encoded by the *pil-chp* operon (*pilGHIJK-chpABC*) in P. aeruginosa (37, 38), and was shown to react to mechanosensing signals upon surface attachment (39). The main components of the system are a transmembrane chemoreceptor (PilJ), a histidine kinase (ChpA), and the response regulators PilG and PilH, all of which share homology with flagellar chemotaxis proteins (40). The response regulators modulate intracellular cAMP levels by regulating the activity of the adenylate cyclase CyaB (41, 42). Increased intracellular cAMP levels subsequently activate the virulence regulator Vfr (43). Importantly, PilG and PilH also regulate TFP dynamics in a cAMP-independent manner by modulating PilB-driven pilus extension and PilT-driven pilus retraction, respectively (37, 41).

In this study, we characterized aspects of the natural competence program of A. baumannii. We showed that the pathogen’s transformability varies significantly during the different growth phases. This variability is due to growth phase-dependent production of its TFP. From genetically engineered mutants and pilus visualization, we demonstrated that pilus-related genes are essential for the bacterium’s transformability and its surface motility, while pilus-unrelated competence genes do not interfere with the motility phenotype. Based on their homology to their P. aeruginosa counterparts, we identified several conserved TFP regulators and showed that these regulators affect cellular piliation status and influence natural transformation in A. baumannii.

## RESULTS AND DISCUSSION

### Transformation of A. baumannii occurs mostly during exponential growth.

To begin, we sought the optimized conditions for transformability for strain A118 (13) (Table 1) by adapting variations from different available protocols. We found the ideal conditions to be after aerobic growth in broth followed by DNA uptake on agar surfaces (see Materials and Methods). We next tested the impact of the growth phase during the liquid culturing time on the bacterial transformability. As shown in Fig. 1B, the highest levels of transformation were observed for bacteria grown to the exponential phase (i.e., ∼90 min), while the strain’s transformability fell below detection after A. baumannii’s dilution into fresh medium or upon entry into the stationary phase (Fig. 1). This behavior is distinct from that of A. baylyi, which can reach transformation frequencies up to 0.7% of all cells in an autotrophy/prototrophy transformation experiment (44) and is transformable throughout all growth phases with varying efficiencies (45, 46). This underscores the importance of studying A. baumannii in its own right and not relying on assumptions made from model bacteria.

**TABLE 1:** Bacterial strains and plasmids used in this study

Strain or plasmid	Genotype or description	Internal strain no.	Source or reference(s) (original strain and genome sequence)
A. baumannii			
A118	Wild type; Amp^r^ Cm^r^; ATCC BAA-2093	MB#5144	ATCC culture collection (13, 80)
A118Δhcp1::kan	A118 with *hcp1* replaced by *aph* cassette, using pGP704-Sac28-Δhcp1::kan (A118Δ*hcp1*::*aph*); Amp^r^ Cm^r^	MB#6403	This study
A118-pilA-FLAG	A118 carrying translational fusion encoding *pilA*-FLAG allele at native pilA locus; Amp^r^ Cm^r^	MB#8859	This study
A118-pilA-FLAGΔpilQ	A118-pilA-FLAG with *pilQ* deleted, using suicide plasmid pGP704-Sac-Kan-ΔpilQ; Amp^r^ Cm^r^	MB#8860	This study
A118ΔpilA	A118 with *pilA* deleted, using suicide plasmid pGP704-Sac-Kan-ΔpilA; Amp^r^ Cm^r^	MB#8585	This study
A118ΔpilQ	A118 with *pilQ* deleted, using suicide plasmid pGP704-Sac-Kan-ΔpilQ; Amp^r^ Cm^r^	MB#8584	This study
A118ΔpilT	A118 with *pilT* deleted, using suicide plasmid pGP704-Sac-Kan-ΔpilT; Amp^r^ Cm^r^	MB#8586	This study
A118ΔpilU	A118 with *pilU* deleted, using suicide plasmid pGP704-Sac-Kan-ΔpilU; Amp^r^ Cm^r^	MB#8861	This study
A118ΔcomEA	A118 with *comEA* deleted, using suicide plasmid pGP704-Sac-Kan-ΔcomEA; Amp^r^ Cm^r^	MB#8862	This study
A118ΔcomF	A118 with *comF* deleted, using suicide plasmid pGP704-Sac-Kan-ΔcomF; Amp^r^ Cm^r^	MB#8863	This study
A118ΔdprA	A118 with *dprA* deleted, using suicide plasmid pGP704-Sac-Kan-ΔdprA; Amp^r^ Cm^r^	MB#8864	This study
A118ΔcomM	A118 with *comM* deleted, using suicide plasmid pGP704-Sac-Kan-ΔcomM; Amp^r^ Cm^r^	MB#8865	This study
A118-TnAraC	A118 containing mini-Tn7-*araC* (TnAraC); Amp^r^ Cm^r^ Gent^r^	MB#8874	This study
A118ΔpilA-TnAraC	A118ΔpilA containing mini-Tn7-*araC* (TnAraC); Amp^r^ Cm^r^ Gent^r^	MB#8875	This study
A118ΔpilA-TnPilA	A118ΔpilA containing mini-Tn7-*araC*-*pilA* (TnPilA); Amp^r^ Cm^r^ Gent^r^	MB#8866	This study
A118ΔpilQ-TnAraC	A118ΔpilQ containing mini-Tn7-*araC* (TnAraC); Amp^r^ Cm^r^ Gent^r^	MB#8876	This study
A118ΔpilQ-TnPilQ	A118ΔpilQ containing mini-Tn7-*araC*-*pilQ* (TnPilQ); Amp^r^ Cm^r^ Gent^r^	MB#8867	This study
A118ΔpilT-TnAraC	A118ΔpilT containing mini-Tn7-*araC* (TnAraC); Amp^r^ Cm^r^ Gent^r^	MB#8877	This study
A118ΔpilT-TnPilT	A118ΔpilT containing mini-Tn7-*araC*-*pilT* (TnPilT); Amp^r^ Cm^r^ Gent^r^	MB#8868	This study
A118ΔpilU-TnAraC	A118ΔpilU containing mini-Tn7-*araC* (TnAraC); Amp^r^ Cm^r^ Gent^r^	MB#8878	This study
A118ΔpilU-TnPilU	A118ΔpilU containing mini-Tn7-*araC*-*pilU* (TnPilU); Amp^r^ Cm^r^ Gent^r^	MB#8869	This study
A118ΔcomEA-TnAraC	A118ΔcomEA containing mini-Tn7-*araC* (TnAraC); Amp^r^ Cm^r^ Gent^r^	MB#8879	This study
A118ΔcomEA-TnComEA	A118ΔcomEA containing mini-Tn7-*araC*-*comEA* (TnComEA); Amp^r^ Cm^r^ Gent^r^	MB#8870	This study
A118ΔcomF-TnAraC	A118ΔcomF containing mini-Tn7-*araC* (TnAraC); Amp^r^ Cm^r^ Gent^r^	MB#8880	This study
A118ΔcomF-TnComF	A118ΔcomF containing mini-Tn7-*araC*-*comF* (TnComF); Amp^r^ Cm^r^ Gent^r^	MB#8871	This study
A118ΔdprA-TnAraC	A118ΔdprA containing mini-Tn7-*araC* (TnAraC); Amp^r^ Cm^r^ Gent^r^	MB#8881	This study
A118ΔdprA-TnDprA	A118ΔdprA containing mini-Tn7-*araC*-*dprA* (TnDprA); Amp^r^ Cm^r^ Gent^r^	MB#8872	This study
A118ΔcomM-TnAraC	A118ΔcomM containing mini-Tn7-*araC* (TnAraC); Amp^r^ Cm^r^ Gent^r^	MB#8882	This study
A118ΔcomM-TnComM	A118ΔcomM containing mini-Tn7-*araC*-*comM* (TnComM); Amp^r^ Cm^r^ Gent^r^	MB#8873	This study
A118ΔpilS	A118 with *pilS* deleted, using suicide plasmid pGP704-Sac-Kan-ΔpilS; Amp^r^ Cm^r^	MB#8590	This study
A118ΔpilR	A118 with *pilR* deleted, using suicide plasmid pGP704-Sac-Kan-ΔpilR; Amp^r^ Cm^r^	MB#8591	This study
A118ΔchpA	A118 with *chpA* deleted, using suicide plasmid pGP704-Sac-Kan-ΔchpA; Amp^r^ Cm^r^	MB#8588	This study
A118ΔpilG	A118 with *pilG* deleted, using suicide plasmid pGP704-Sac-Kan-ΔpilG; Amp^r^ Cm^r^	MB#8587	This study
A118ΔpilH	A118 with *pilH* deleted, using suicide plasmid pGP704-Sac-Kan-ΔpilH; Amp^r^ Cm^r^	MB#8589	This study
A118ΔpilS-TnAraC	A118ΔpilS containing mini-Tn7-*araC* (TnAraC); Amp^r^ Cm^r^ Gent^r^	MB#8888	This study
A118ΔpilS-TnPilS	A118ΔpilS containing mini-Tn7-*araC*-*pilS* (TnPilS); Amp^r^ Cm^r^ Gent^r^	MB#8883	This study
A118ΔpilR-TnAraC	A118ΔpilR containing mini-Tn7-*araC* (TnAraC); Amp^r^ Cm^r^ Gent^r^	MB#8889	This study
A118ΔpilR-TnPilR	A118ΔpilR containing mini-Tn7-*araC*-*pilR* (TnPilR); Amp^r^ Cm^r^ Gent^r^	MB#8884	This study
A118ΔchpA-TnAraC	A118ΔchpA containing mini-Tn7-*araC* (TnAraC); Amp^r^ Cm^r^ Gent^r^	MB#8890	This study
A118ΔchpA-TnChpA	A118ΔchpA containing mini-Tn7-*araC*-*chpA* (TnChpA); Amp^r^ Cm^r^ Gent^r^	MB#8885	This study
A118ΔpilG-TnAraC	A118ΔpilG containing mini-Tn7-*araC* (TnAraC); Amp^r^ Cm^r^ Gent^r^	MB#8891	This study
A118ΔpilG-TnPilG	A118ΔpilG containing mini-Tn7-*araC*-*pilG* (TnPilG); Amp^r^ Cm^r^ Gent^r^	MB#8886	This study
A118ΔpilH-TnAraC	A118ΔpilH containing mini-Tn7-*araC* (TnAraC); Amp^r^ Cm^r^ Gent^r^	MB#8892	This study
A118ΔpilH-TnPilH	A118ΔpilH containing mini-Tn7-*araC*-*pilH* (TnPilH); Amp^r^ Cm^r^ Gent^r^	MB#8887	This study
A118-pilA-FLAGΔpilS	A118-pilA-FLAG with *pilS* deleted, using suicide plasmid pGP704-Sac-Kan-ΔpilS; Amp^r^ Cm^r^	MB#8893	This study
A118-pilA-FLAGΔpilR	A118-pilA-FLAG with *pilR* deleted, using suicide plasmid pGP704-Sac-Kan-ΔpilR; Amp^r^ Cm^r^	MB#8894	This study
A118-pilA-FLAGΔchpA	A118-pilA-FLAG with *chpA* deleted, using suicide plasmid pGP704-Sac-Kan-ΔchpA; Amp^r^ Cm^r^	MB#8895	This study
A118-pilA-FLAGΔpilG	A118-pilA-FLAG with *pilG* deleted, using suicide plasmid pGP704-Sac-Kan-ΔpilG; Amp^r^ Cm^r^	MB#8896	This study
A118-pilA-FLAGΔpilH	A118-pilA-FLAG with *pilH* deleted, using suicide plasmid pGP704-Sac-Kan-ΔpilH; Amp^r^ Cm^r^	MB#8897	This study
A118-pilA(A61C)	A118 with site-directed point mutation in *pilA* [resulting in PilA(A61C)]; Amp^r^ Cm^r^	MB#8898	This study
A118-pilA(G60C)	A118 with site-directed point mutation in *pilA* [resulting in PilA(G60C)]; Amp^r^ Cm^r^	MB#8918	This study
A118-pilA(V62C)	A118 with site-directed point mutation in *pilA* [resulting in PilA(V62C)]; Amp^r^ Cm^r^	MB#8919	This study
A118-pilA(T64C)	A118 with site-directed point mutation in *pilA* [resulting in PilA(T64C)]; Amp^r^ Cm^r^	MB#8920	This study
A118-pilA(S67C)	A118 with site-directed point mutation in *pilA* [resulting in PilA(S67C)]; Amp^r^ Cm^r^	MB#8921	This study
A118-pilA(T72C)	A118 with site-directed point mutation in *pilA* [resulting in PilA(T72C)]; Amp^r^ Cm^r^	MB#8922	This study
A118-pilA(T75C)	A118 with site-directed point mutation in *pilA* [resulting in PilA(T75C)]; Amp^r^ Cm^r^	MB#8923	This study
A118-pilA(A61C)ΔpilS	A118-pilA(A61C) with *pilS* deleted, using suicide plasmid pGP704-Sac-Kan-ΔpilS; Amp^r^ Cm^r^	MB#8899	This study
A118-pilA(A61C)ΔpilR	A118-pilA(A61C) with *pilR* deleted, using suicide plasmid pGP704-Sac-Kan-ΔpilR; Amp^r^ Cm^r^	MB#8900	This study
A118-pilA(A61C)ΔchpA	A118-pilA(A61C) with *chpA* deleted, using suicide plasmid pGP704-Sac-Kan-ΔchpA; Amp^r^ Cm^r^	MB#8901	This study
A118-pilA(A61C)ΔpilG	A118-pilA(A61C) with *pilG* deleted, using suicide plasmid pGP704-Sac-Kan-ΔpilG; Amp^r^ Cm^r^	MB#8902	This study
A118-pilA(A61C)ΔpilH	A118-pilA(A61C) with *pilH* deleted, using suicide plasmid pGP704-Sac-Kan-ΔpilH; Amp^r^ Cm^r^	MB#8903	This study
A118-pilA(A61C)ΔpilT	A118-pilA(A61C) with *pilT* deleted, using suicide plasmid pGP704-Sac-Kan-ΔpilT; Amp^r^ Cm^r^	MB#8904	This study
A118-pilA(A61C)ΔpilTΔpilS	A118-pilA(A61C)ΔpilT with *pilS* deleted, using suicide plasmid pGP704-Sac-Kan-ΔpilS; Amp^r^ Cm^r^	MB#8905	This study
A118-pilA(A61C)ΔpilTΔpilR	A118-pilA(A61C)ΔpilT with *pilR* deleted, using suicide plasmid pGP704-Sac-Kan-ΔpilR; Amp^r^ Cm^r^	MB#8906	This study
A118-pilA(A61C)ΔpilTΔchpA	A118-pilA(A61C)ΔpilT with *chpA* deleted, using suicide plasmid pGP704-Sac-Kan-ΔchpA; Amp^r^ Cm^r^	MB#8907	This study
A118-pilA[A61C] ΔpilTΔpilG	A118-pilA(A61C)ΔpilT with *pilG* deleted, using suicide plasmid pGP704-Sac-Kan-ΔpilG; Amp^r^ Cm^r^	MB#8908	This study
A118-pilA(A61C)ΔpilTΔpilH	A118-pilA(A61C)ΔpilT with *pilH* deleted, using suicide plasmid pGP704-Sac-Kan-ΔpilH; Amp^r^ Cm^r^	MB#8909	This study
A118ΔpilR-TnPilR-D56E	A118ΔpilR containing mini-Tn7-*araC*-*pilR*(D56E) [TnPilR(D56E)]; Amp^r^ Cm^r^ Gent^r^	MB#8910	This study
A118ΔpilSR	A118 with *pilSR* deleted, using suicide plasmid pGP704-Sac-Kan-ΔpilSR; Amp^r^ Cm^r^	MB#8911	This study
A118ΔpilSR-TnAraC	A118 ΔpilSR containing mini-Tn7-*araC* (TnAraC); Amp^r^ Cm^r^ Gent^r^	MB#8912	This study
A118ΔpilSR-TnPilR	A118ΔpilSR containing mini-Tn7-*araC*-*pilR* (TnPilR); Amp^r^ Cm^r^ Gent^r^	MB#8913	This study
A118ΔpilSR-TnPilR(D56E)	A118ΔpilSR containing mini-Tn7-*araC*-*pilR*(D56E) [TnPilR(D56E)]; Amp^r^ Cm^r^ Gent^r^	MB#8914	This study
A118-pilA(A61C)ΔpilTΔpilR-TnAraC	A118-pilA(A61C)ΔpilTΔpilR containing mini-Tn7-*araC* (TnAraC); Amp^r^ Cm^r^ Gent^r^	MB#8915	This study
A118-pilA(A61C)ΔpilTΔpilR-TnPilR	A118-pilA(A61C)ΔpilTΔpilR containing mini-Tn7-*araC*-*pilR* (TnPilR); Amp^r^ Cm^r^ Gent^r^	MB#8916	This study
A118-pilA(A61C)ΔpilTΔpilR-TnPilR(D56E)	A118-pilA(A61C)ΔpilTΔpilR containing mini-Tn7-*araC*-*pilR*(D56E) [TnPilR(D56E)]; Amp^r^ Cm^r^ Gent^r^	MB#8917	This study
E. coli S17-1λpir	Tp^r^ Sm^r^ *recA* *thi* *pro* *hsdR2M1* RP4:2-Tc:Mu:Km^r^ Tn7 (λpir)	MB#648	81
Plasmids			
pGP704-Sac28	Suicide plasmid, *oriR6K sacB* Amp^r^	MB#649	71
pGP704-Sac-Kan	Suicide plasmid, *oriR6K sacB* Kan^r^	MB#6038	72
pGP704-TnAraC	pGP704 with mini-Tn7 carrying *araC* and P_BAD_; Amp^r^ Gent^r^	MB#5513	23, 63
pUX-BF-13	*oriR6K*, helper plasmid with Tn7 transposition function; Amp^r^	MB#457	74
pGP704-Sac28-Δhcp::kan	pGP704-Sac28 carrying *hcp* with an insertion of *aph;* Amp^r^ Kan^r^	MB#8924	This study
pGP704-Sac-Kan-pilA-FLAG	pGP704-Sac-Kan carrying the *pilA*-FLAG allele with flanking regions; Kan^r^	MB#8925	This study
pGP704-Sac-Kan-ΔpilQ	pGP704-Sac-Kan carrying a deletion within *pilQ;* Kan^r^	MB#8926	This study
pGP704-Sac-Kan-ΔpilA	pGP704-Sac-Kan carrying a deletion within *pilA*; Kan^r^	MB#8927	This study
pGP704-Sac-Kan-ΔpilT	pGP704-Sac-Kan carrying a deletion within *pilT*; Kan^r^	MB#8928	This study
pGP704-Sac-Kan-ΔpilU	pGP704-Sac-Kan carrying a deletion within *pilU;* Kan^r^	MB#8929	This study
pGP704-Sac-Kan-ΔcomEA	pGP704-Sac-Kan carrying a deletion within *comEA*; Kan^r^	MB#8930	This study
pGP704-Sac-Kan-ΔcomF	pGP704-Sac-Kan carrying a deletion within *comF*; Kan^r^	MB#8931	This study
pGP704-Sac-Kan-ΔdprA	pGP704-Sac-Kan carrying a deletion within *dprA*; Kan^r^	MB#8932	This study
pGP704-Sac-Kan-ΔcomM	pGP704-Sac-Kan carrying a deletion within *comM*; Kan^r^	MB#8933	This study
pGP704-TnPilA	pGP704 with mini-Tn7 carrying *araC* and P_BAD_-driven *pilA*; Amp^r^ Gent^r^	MB#8934	This study
pGP704-TnPilQ	pGP704 with mini-Tn7 carrying *araC* and P_BAD_-driven *pilQ*; Amp^r^ Gent^r^	MB#8935	This study
pGP704-TnPilT	pGP704 with mini-Tn7 carrying *araC* and P_BAD_-driven *pilT*; Amp^r^ Gent^r^	MB#8936	This study
pGP704-TnPilU	pGP704 with mini-Tn7 carrying *araC* and P_BAD_-driven *pilU*; Amp^r^ Gent^r^	MB#8937	This study
pGP704-TnComEA	pGP704 with mini-Tn7 carrying *araC* and P_BAD_-driven *comEA*; Amp^r^ Gent^r^	MB#8938	This study
pGP704-TnComF	pGP704 with mini-Tn7 carrying *araC* and P_BAD_-driven *comF*; Amp^r^ Gent^r^	MB#8939	This study
pGP704-TnDprA	pGP704 with mini-Tn7 carrying *araC* and P_BAD_-driven *dprA*; Amp^r^ Gent^r^	MB#8940	This study
pGP704-TnComM	pGP704 with mini-Tn7 carrying *araC* and P_BAD_-driven *comM*; Amp^r^ Gent^r^	MB#8941	This study
pGP704-Sac-Kan-ΔpilS	pGP704-Sac-Kan carrying a deletion within *pilS*; Kan^r^	MB#8942	This study
pGP704-Sac-Kan-ΔpilR	pGP704-Sac-Kan carrying a deletion within *pilR*; Kan^r^	MB#8943	This study
pGP704-Sac-Kan-ΔchpA	pGP704-Sac-Kan carrying a deletion within *chpA*; Kan^r^	MB#8944	This study
pGP704-Sac-Kan-ΔpilG	pGP704-Sac-Kan carrying a deletion within *pilG*; Kan^r^	MB#8945	This study
pGP704-Sac-Kan-ΔpilH	pGP704-Sac-Kan carrying a deletion within *pilH*; Kan^r^	MB#8946	This study
pGP704-TnPilS	pGP704 with mini-Tn7 carrying *araC* and P_BAD_-driven *pilS*; Amp^r^ Gent^r^	MB#8947	This study
pGP704-TnPilR	pGP704 with mini-Tn7 carrying *araC* and P_BAD_-driven *pilR*; Amp^r^ Gent^r^	MB#8948	This study
pGP704-TnChpA	pGP704 with mini-Tn7 carrying *araC* and P_BAD_-driven *chpA*; Amp^r^ Gent^r^	MB#8949	This study
pGP704-TnPilG	pGP704 with mini-Tn7 carrying *araC* and P_BAD_-driven *pilG*; Amp^r^ Gent^r^	MB#8950	This study
pGP704-TnPilH	pGP704 with mini-Tn7 carrying *araC* and P_BAD_-driven *pilH*; Amp^r^ Gent^r^	MB#8951	This study
pGP704-Sac-Kan-pilA(A61C)	pGP704-Sac-Kan carrying a genome fragment resulting in a site-directed point mutation in *pilA* [resulting in PilA(A61C)]; Kan^r^	MB#8952	This study
pGP704-TnPilR(D56E)	pGP704 with mini-Tn7 carrying *araC* and P_BAD_-driven *pilR*(D56E); Amp^r^ Gent^r^	MB#8953	This study
pGP704-Sac-Kan-delta-pilSR	pGP704-Sac-Kan carrying a deletion within *pilSR;* Kan^r^	MB#8954	This study
pGP704-Sac-Kan-pilA(G60C)	pGP704-Sac-Kan carrying a genome fragment resulting in a site-directed point mutation in *pilA* [resulting in PilA(G60C)]; Kan^r^	MB#8955	This study
pGP704-Sac-Kan-pilA(V62C)	pGP704-Sac-Kan carrying a genome fragment resulting in a site-directed point mutation in *pilA* [resulting in PilA(V62C)]; Kan^r^	MB#8956	This study
pGP704-Sac-Kan-pilA(T64C)	pGP704-Sac-Kan carrying a genome fragment resulting in a site-directed point mutation in *pilA* [resulting in PilA(T64C)]; Kan^r^	MB#8957	This study
pGP704-Sac-Kan-pilA(S67C)	pGP704-Sac-Kan carrying a genome fragment resulting in a site-directed point mutation in *pilA* [resulting in PilA(S67C)]; Kan^r^	MB#8958	This study
pGP704-Sac-Kan-pilA(T72C)	pGP704-Sac-Kan carrying a genome fragment resulting in a site-directed point mutation in *pilA* [resulting in PilA(T72C)]; Kan^r^	MB#8959	This study
pGP704-Sac-Kan-pilA(T75C)	pGP704-Sac-Kan carrying a genome fragment resulting in a site-directed point mutation in *pilA* [resulting in PilA(T75C)]; Kan^r^	MB#8960	This study

To better understand this rather tight transformation window, we scored transcript levels of selected competence genes as a function of time. To identify competence genes in A. baumannii strain A118, we investigated its previously published genome sequence, where Ramirez et al. reported 186 scaffolds at least 500 bp in length (47). However, the overall high number of contigs (1,647 contigs, accession number AEOW01000000) complicated the search for genes and the determination of operons. Therefore, we resequenced the strain using long-read PacBio technology and *de novo* assembled its genome (see Materials and Methods).

With the 3,750,370 bp closed genome of A. baumannii at hand (Table 2), we then identified all known competence genes based on homology to the competent model organisms A. baylyi and V. cholerae (21, 48) (Table 3). Next, we scored the transcript levels of six selected competence genes using qRT-PCR: *pilA*, *pilQ*, *pilT*, *comEA*, *comEC*, and *dprA.* The encoded products of these genes (e.g., the major pilin subunit PilA, the outer membrane secretin PilQ, and the retraction ATPase PilT) contribute to the assembly of the central TFP of the DNA uptake machinery and are key for DNA uptake in Gram-negative bacteria. They are also important for DNA translocation across the outer (ComEA) and inner (ComEC) membrane or in recombination-mediating activity (DprA) inside the cytosol (7) (Fig. 1A). As shown in Fig. 1C, the transcript levels varied at time points before, during, and after the growth phase in which transformation levels were the highest (Fig. 1B). In general, the *pil* genes specifically peaked around 90 min after dilution (Fig. 1C), which correlated with the beginning of the transformation window (Fig. 1B). This differed from previous reports on A. baylyi where Porstendörfer et al. showed that the expression of the *pilA* homologues major pilin gene *comP* decreased immediately after dilution into fresh medium, with the lowest levels in the mid-exponential phase. In their study, maximal *comP* expression levels were observed in the late stationary phase (46).

**TABLE 2:** Statistics on PacBio genome sequencing data and assembly

Parameter	A. baumannii A118 result
Internal strain ID	MB#5144
BioSample ID	SAMN15507634
GenBank accession no.	CP059039
No. of bases	662,248,721 bp
No. of reads	39,350
Mean read length	16,829 bp
Total no. of contigs	1
Maximum contig length	3,787,003 bp
Contig length after circularization	3,750,370 bp
Total genome size	3,750,370 bp
Mean coverage	163×
GC content	39.1%

**TABLE 3:** Competence and TFP-related genes in strain A118

Gene name^*a*^	Locus tag in A118^*b*^ (H0N27_XXXXX)	Automatically annotated gene product in A118^*b*^	Homolog (locus tag) in^*c*^:		
			V. cholerae N16961 [VC(A)XXXX]	A. baylyi ADP1 (ACIADXXXX)	P. aeruginosa PAO1 (PAXXXX)
Type IV pilus					
*pilM*	01440	Pilus assembly protein PilM	*pilM* (2634)	*pilM* (3360)	*pilM* (5044)
*pilN*	01445	*pilN* domain-containing protein	*pilN* (2633)	*pilN* (3359)	*pilN* (5043)
*pilO*	01450	Type IV-A pilus biogenesis protein PilO	*pilO* (2632)	*pilO* (3357)	*pilO* (5042)
*pilP*	01455	Pilus assembly protein PilP	*pilP* (2631)	*pilP* (3356)	*pilP* (5041)
*pilQ*	01460	Type IV pilus secretin PilQ family protein	*pilQ* (2630)	*pilQ* (3355)	*pilQ* (5040)
*pilT*	13360	Type IV pilus twitching motility protein PilT	*pilT* (0462)	*pilT* (0912)	*pilT* (0395)
*pilU*	13365	PilT/PilU family type 4a pilus ATPase	*pilU* (0463)	*pilU* (0911)	*pilU* (0396)
*pilF*	14985	Type IV pilus biogenesis/stability protein PilW	*pilF* (1612)	*pilF* (0558)	*pilF* (3805)
*fimV*	15350	Hypothetical protein			*fimV* (3115)
*pilA*	01510	Pilin	*pilA* (2423)	comP (3338)	*pilA* (4525)
*pilB*	15830	Type IV-A pilus assembly ATPase PilB	*pilB* (2424)	*pilB* (0362)	*pilB* (4526)
*pilC*	15835	Type II secretion system F family protein	*pilC* (2425)	*pilC* (0361)	*pilC* (4527)
*pilD*	15840	Prepilin peptidase	*pilD* (2426)	*pilD* (0360)	*pilD* (4528)
*tsaP*	16565	LysM peptidoglycan-binding domain-containing protein	*tsaP* (0047)	0210	0020
*fimU*	01560	GspH/FimT family pseudopilin	VC0858 (part of minor pilin cluster [21])	*fimU* (3321)	*fimU* (4550)
*pilV*	01565	Type IV pilus modification protein PilV		*pilV* (3319)	*pilV* (4551)
*pilW*	01570	PilW family protein		*comB* (3318)	*pilW* (4552)
*pilX*	01575	Pilus assembly protein		*pilX* (3317)	
*pilY1*	01580	VWA domain-containing protein		*comC* (3316)	*pilY1* (4554)
Gene name to be defined	01585	Prepilin-type N-terminal cleavage/methylation domain-containing protein		*comE* (3315)	
*pilE*	01590	Prepilin-type N-terminal cleavage/methylation domain-containing protein	*VC0857* (part of minor pilin cluster [21])	*comF* (3314)	*pilE* (4556)
*fimT*	02975	GspH/FimT family pseudopilin		*fimT* (0695)	*fimT* (4549)
DNA uptake & translocation					
*comEA*	14695	ComEA family DNA-binding protein	*comEA* (1917)	*comEA* (3064)	3140
*comEC*	04190	DNA internalization-related competence protein ComEC/Rec2	*comEC* (1879)	*comA* (2639)	*comEC* (2984)
*comF*	01930	ComF family protein	*comF* (2719)	*comF* (3236)	*comF* (0489)
ssDNA binding & recombination					
*ssb*	00985	Single-stranded DNA-binding protein	*ssb* (0397)	*ssb* (3449)	*ssb* (4232)
*dprA*	16570	DNA-protecting protein DprA	*dprA* (0048)	0309	0021
*recA*	07425	Recombinase RecA	*recA* (0543)	*recA* (1385)	*recA* (3617)
*comM*	16365	YifB family Mg chelatase-like AAA ATPase	*comM* (0032)	0242	5290
TFP regulation					
*pilS*	16280	PAS domain-containing sensor histidine kinase		*pilS* (0259)	*pilS* (4546)
*pilR*	16285	Sigma-54-dependent Fis family transcriptional regulator		*pilR* (0258)	*pilR* (4547)
*pilG*	03110	Twitching motility response regulator PilG		*pilG* (0786)	*pilG* (0408)
*pilH*	03115	Response regulator		*pilH* (0787)	*pilH* (0409)
*pilI*	03120	Purine-binding chemotaxis protein CheW		*pilI* (0788)	*pilI* (0410)
*pilJ*	03125	Methyl-accepting chemotaxis protein		*pilJ* (0789)	*pilJ* (0411)
Nonexistent					*pilK* (0412)
*chpA*	03130	Hpt domain-containing protein		*chpA* (0790)	*chpA* (0413)
To be defined	03135	Hypothetical protein (predicted as cheA-like protein by HHpred)			
Nonexistent					*chpB* (0414)
Nonexistent					*chpC* (0415)
Nonexistent					*chpD* (0416)
Nonexistent					*chpE* (0417)
*cyaB*	09340	Adenylate/guanylate cyclase domain-containing protein		1397	*cyaB* (3217)
To be defined	11735	cAMP-activated global transcriptional regulator CRP	*crp* (2614)	*vfr* (1262)	*vfr* (0652)
fimL	12210	Chemotaxis protein		1136	*fimL* (1822)
Other genes					
*glmS*	00430	Glutamine-fructose-6-phosphate transaminase (isomerizing)	mTn*7* insertion site downstream of *glmS*		
*hcp*	11090	Type VI secretion system tube protein Hcp	*aph* (Kan^r^) cassette inserted in *hcp* in tDNA		

a A118 gene names are according to homologs described in references 61 and 82.

b Locus tags of strain A118 and automatic annotations are according to accession number CP059039 of this study.

c Homologs were determined using PATRIC BLAST and are based on protein sequence similarities, the positions of genes in operons, and their predicted functions. Only significant BLAST hits are shown (E value <0.01). Locus tags of strain N16961 [VC(A)XXXX] are according to accession numbers NC_002505 and NC_002506 (83). Locus tags of strain ADP1 (ACIADXXXX) are according to accession number NC_005966 (84). Locus tags of strain PAO1 (PAXXXX) are according to accession number NC_002516 (85).

### Peak transformation coincides with type IV pilin production.

As transcript levels do not necessarily reflect the cellular protein levels, we next tested the production of the major pilin protein PilA. To accomplish this, we translationally fused PilA to a short FLAG tag, which gave a strain with the *pilA*-FLAG allele at the native *pilA* locus that retained suboptimal but still high transformation levels (Fig. 1D). We grew this genetically engineered strain and quantified the PilA-FLAG protein levels in cells over time by Western blotting. From these data, we concluded that no PilA-FLAG was detected early on in growth. We observed that the major pilin level peaked at approximately 90 to 105 min postdilution (optical density at 600 nm [OD_600_] of ∼0.6 to 0.7), declined after 120 min of growth (OD_600_ of ∼1.0), and ultimately disappeared later during growth (Fig. 1E). To control for surface-exposed pili that might have been lost through shearing during the harvesting process, we repeated the same experiment in a *pilQ* deletion strain in which pili cannot cross the outer membrane. We observed a similar protein production pattern over time (Fig. 1F) to that of the parental wild-type (WT) background strain (Fig. 1E). From these data, we concluded that A. baumannii produces its TFP solely during the early exponential phase under liquid growth conditions. This is in stark contrast to A. baylyi, in which the major pilin is primarily produced in the stationary phase and is either absent or only present at low levels during the exponential growth phase (46).

### Pilus visualization elucidates diverse phenotypes.

**FIG 2:**
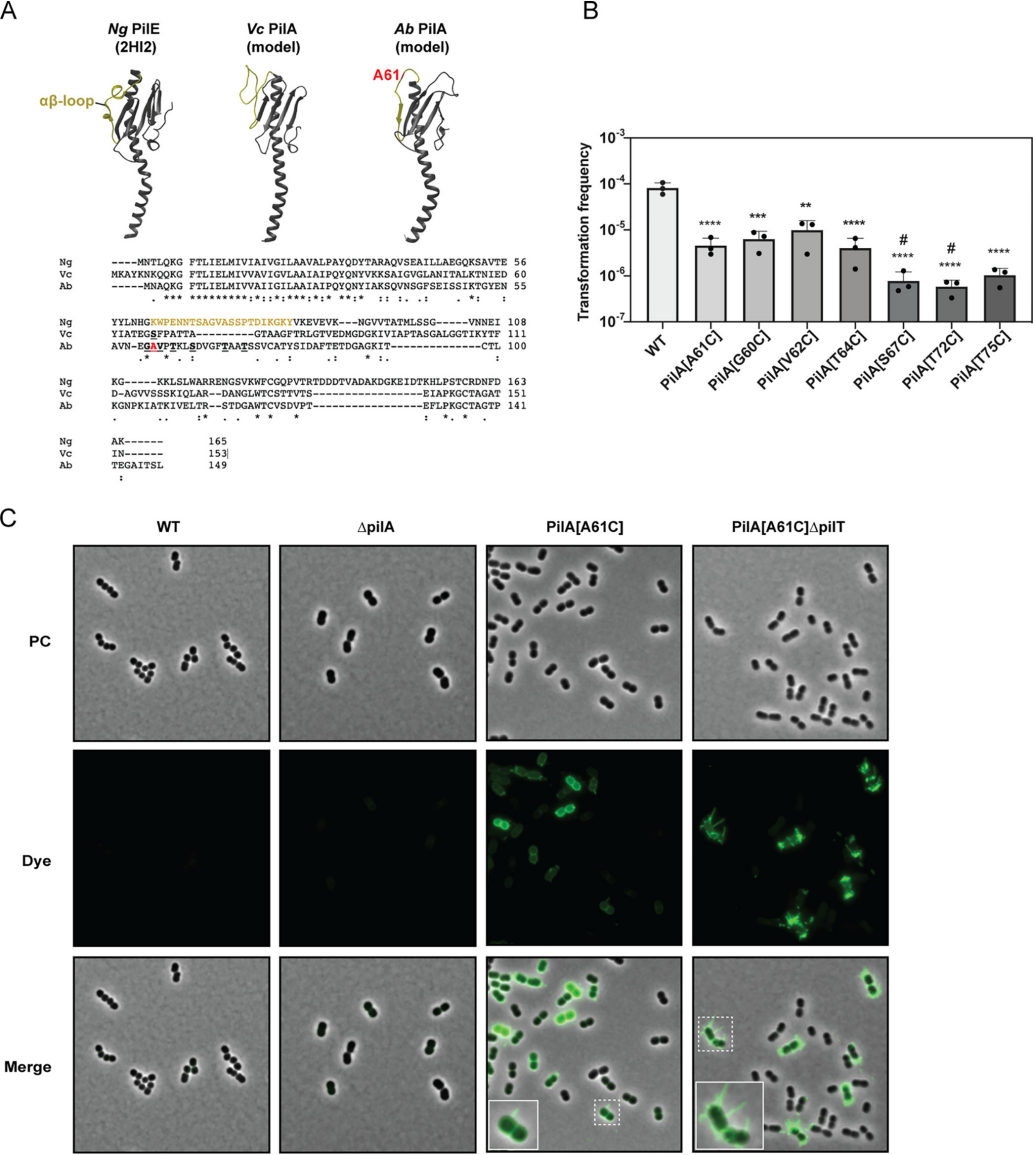
Design and functionality of PilA cysteine knock-in variants. (A) 3D structural model of the Neisseria gonorrhoeae major type IV pilin PilE (PDB, 2HI2 [78]), which is shown alongside Phyre2 (51) structural predictions of the major pilin PilA of pandemic V. cholerae and of PilA of A. baumannii strain A118 (this study). The conserved αβ-loops are shown in greenish yellow, and the residue chosen for the cysteine exchange (A61) is shown in red. Bottom panel: sequence alignments of N. gonorrhoeae PilE (Ng; Uniprot P02974), V. cholerae PilA (Vc; protein ID AWB74893.1 [79]), and A. baumannii PilA (Ab; strain A118 and protein ID H0N27_01510) using Clustal Omega. The αβ-loop is colored in yellow. The functional cysteine substitution in V. cholerae’s PilA is highlighted (S67 [23]). Residues tested in A. baumannii in this study are shown underlined. (B) Natural transformability of PilA cysteine knock-in variants. Bars show the average transformation frequency of three independent experiments (±SD). Statistical analyses were performed on log-transformed data using a one-way ANOVA followed by Sidak’s multiple-comparison test. Each mutant strain is compared to the WT strain. #, under detection limit in at least one experiment, in which case the detection limit was used for the calculation of the average value and statistical analyses. **, *P* < 0.01; ***, *P* < 0.001; ****, *P* < 0.0001. (C) Pilus imaging using a thiol-reactive maleimide dye. Snapshot images of A118-*pilA*(A61C), A118-*pilA*(A61C)Δ*pilT*, and the parental WT strain. The Δ*pilA* strain served as an additional negative control. The bacteria were stained with AF-488-Mal and imaged in the phase contrast (PC) or green fluorescence (Dye) channels. A merged image of both channels is shown in the bottom row (Merge). The contrast of the merged images was adjusted for best pilus visualization. An enlargement of the marked region (dotted boxes) is shown as an inset.

We extended our study beyond quantification of the major pilin subunit by visualizing the TFP of A. baumannii. To do so, we aimed at using thiol-reactive maleimide-conjugated dyes to cysteine-containing cell appendages, an approach that has been used for the flagellum of Bacillus subtilis (49) and the tight adherence (Tad) pilus of Caulobacter crescentus (50). To determine the proper location for a site-directed cysteine knock-in into the major pilin PilA, we followed the same protocol as that described for V. cholerae (23). Briefly, we predicted the surface-exposed αβ-loop of PilA using the Phyre2 web portal (51) (Fig. 2A) and found that the overall structure prediction for A. baumannii’s PilA was similar to that of V. cholerae (Fig. 2A) by length and homology in the protein’s N-terminal region. From this analysis, we selected an alanine-to-cysteine mutation at position 61 (A61C), which is comparable to the cysteine knock-in mutant we had previously determined for V. cholerae [PilA(S67C)] (23, 52). We tested the transformation efficiency of the new A118-PilA(A61C) strain relative to that of the parental WT strain. As shown in Fig. 2B, the strain’s transformation efficiency was reduced by approximately 18-fold compared to that of the WT, but it nonetheless maintained robust transformability. To potentially improve the transformation efficiency of cysteine knock-in mutants, we next designed and tested six additional site-directed mutants with amino acid exchanges in the predicted surface-exposed region (G60C, V62C, T64C, T72C, T75C, and S77C). None of these variants resulted in significantly higher transformation levels compared to those of the strain producing PilA(A61C) (Fig. 2B). Therefore, we proceeded with the PilA(A61C) strain given that our previous work on V. cholerae had identified this region as appropriate for the pilus labeling process (23).

To visualize the cells’ pili, we next grew the PilA(A61C) strain to an optical density of ∼0.6 to 0.7, as pilin levels were highest at this growth stage (Fig. 1E). At this time point, we added the thiol-reactive dye (Alexa Fluor 488 C5 maleimide) to the culture and then incubated, washed, and mounted the bacteria onto agarose pads. As shown in Fig. 2C, the bacteria produced pili that, for some cells, extended far beyond the outer membrane. Notably, such extended pili were not restricted to one side of the cell body as recently described for A. baylyi in a preprinted study (53). The bodies of the cells were also stained by the dye in a significant fraction of the population, which suggested incorporation of the pilin subunit into the inner membrane. Another subfraction appeared as fully unlabeled.

We considered two possible explanations for this unlabeled fraction. First, production of the pilus is a heterogeneous and potentially bistable phenotype in A. baumannii similar to competence development in B. subtilis (54–56). Second, the absence of cell body labeling might be due to the production of pilus proteins that were not surface-exposed during the short staining time window. While we previously demonstrated for V. cholerae that cysteine-containing PilA subunits in the inner membrane were labeled even in the absence of the residual TFP components (23), this was not the case for C. crescentus (50). In C. crescentus, only extended pili and membrane-incorporated pilin subunits from pilus retraction events were labeled. It was argued that this might be due to the size-based exclusion of the AF488-maleimide dye by the outer membrane (50). While outer membrane permeability and/or porins might differ between V. cholerae, C. crescentus, and A. baumannii, we speculated that in the latter two organisms, cell-surrounding capsular polysaccharides might also impede the dye’s access to the inner membrane-incorporated pilin subunits. These capsular polysaccharides are considered a major virulence determinant of A. baumannii (57) and could explain the lack of cell body staining in our images. Upon further inspection of the A118 genome sequence, we identified a 22,663 bp genome stretch between the genes *fkpA* and *lldP*, which usually flank capsular biosynthesis clusters (57). BLAST analysis of this cluster showed a 100% conservation of the gene order (18 genes in total) and a 99.2% pairwise sequence identity to the KL51 capsule biosynthesis gene cluster of A. baumannii isolate WM98c (GenBank accession number MN148384 [58]), suggesting that strain A118 is indeed encapsulated.

To test our second hypothesis, we imaged a *pilT*-mutant derivative of strain A118-PilA(A61C). Our underlying rationale was that pili that did extend would remain surface-exposed in this strain in the absence of the retraction ATPase PilT. Indeed, we observed many cells with multiple pili or bundles/clusters thereof (Fig. 2C). Interestingly, the cell bodies themselves were not labeled, which is consistent with the notion that pilus retraction was required to reinsert labeled pilin subunits into the inner membrane after they were pulled through the capsular material and the outer membrane secretin PilQ. However, this strain still showed labeling heterogeneity, which suggested that the pilus production was a phenotype in only a subfraction of the population. Interestingly, A. baumannii strains are known for a number of phase-variable controlled phenotypes, including, for strain AB5075, cell morphology, biofilm formation, and surface motility (59), which is frequently linked to pili. Thus, we can speculate that the pilus-producing and nonproducing bacteria within the population are phase variants that foster diverse phenotypes.

While *pilT* mutants are frequently hyperpiliated (25), including a *pilT* mutant tested for A. nosocomialis M2 (15), the discovery of this in A. baumannii was somewhat surprising, as previous work noted the absence of pili in a *pilTU* mutant of strain ATCC 17978 (60). However, we must note that the absence of pili was scored by transmission electron microscopy in that study, which does not distinguish between diverse cell appendages, and that the fixation process might shear off pili. Interestingly, previous work had demonstrated that other A. baumannii strains produced both thin (∼4 nm wide) and thick (∼7 nm) pili (14). Notably, Wilharm et al. demonstrated that the thick pili were rarely observed on WT cells (only ∼1 in 25 to 50 cells exposed such a pilus) though were frequently witnessed in a *pilT* deletion strain where each cell exposed one or several pili (14). These findings are consistent with the hyperpiliation phenotype described in this study. Indeed, our preliminary scanning electron micrographs of strain A118 supported the observation of multiple cell appendages of different widths (some of those even in a *pilA*-minus strain), which motivated us to adapt the pilus-specific thiol-labeling approach to unambiguously score only PilA-composed TFP.

### Competence and pili mutants are impaired in transformation.

**FIG 3:**
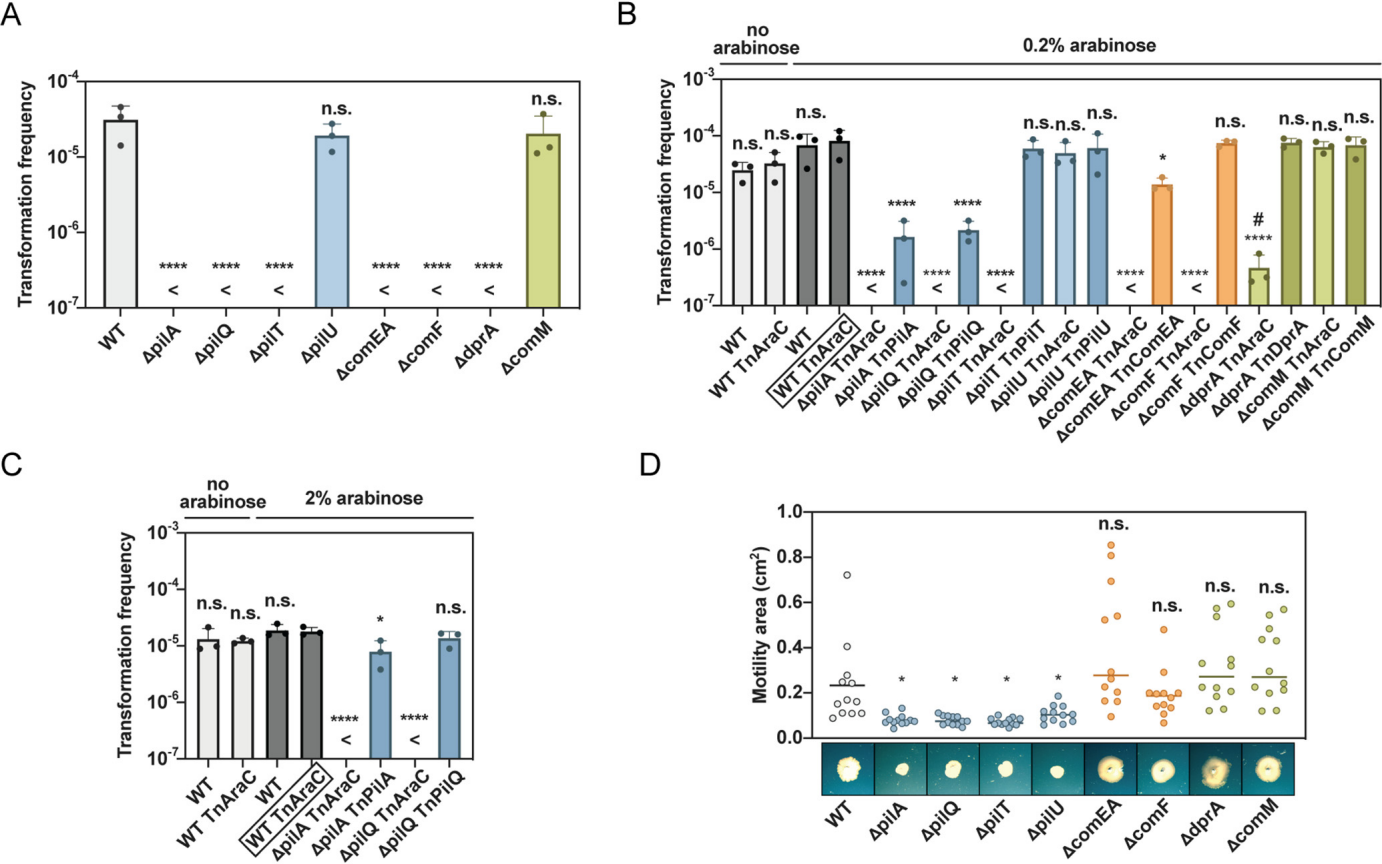
Type IV pilus genes are essential for transformation and surface motility. (A to C) Transformation frequencies of defined mutants (details as in Fig. 1). For complementation, the strains carried a transposon without (control, TnAraC; no gene downstream of P_BAD_ promoter) or with (TnXXXX) the complementing gene on their chromosome and were grown in the absence or presence of 0.2% (B) or 2% (C) arabinose. For all bar plots, transformation frequencies of three independent experiments are plotted as mean values (±SD). <, below detection limit (DL); #, under DL in at least one replicate (DL used for calculation of mean value). Log-transformed data were used for statistical analysis. When no transformants were obtained, the mean of the detection limit was used for statistical analyses. (D) TFP mutants are nonmotile on solid surfaces. Surface motility of the mutants described in panel A is depicted on the *y* axis based on the occupied area on the motility plates. Four biological experiments with three technical replicates are shown for each strain (*n* = 12). Images from one experimental set are shown below the graph. Statistical analyses: panels A to C, one-way ANOVA, using Sidak’s multiple comparisons test; panel D, Brown-Forsythe and Welch ANOVA tests with unpaired *t* test with Welch’s correction. *, *P* < 0.05; ****; *P* < 0.0001; n.s., not significant. The strains were compared to the WT (A and D) or the most appropriate control strain (boxed strain name in panels B and C).

To further investigate the link between pilus production and the strain’s natural transformability, we next generated a set of defined deletion strains. In this context, we also generated knockout strains of conserved competence genes that we considered to encode parts of the DNA uptake machinery based on common knowledge in other naturally competent bacteria (7, 61). Precisely, we generated mutants where the gene products were required for the main steps of the DNA uptake and recombination process, namely, the dynamic TFP (*pilA*, *pilQ*, *pilT*, and *pilU*), DNA uptake and translocation (*comEA* and *comF*), and ssDNA binding and recombination (*dprA* and *comM*) (Fig. 1A and Table 3). We tested these mutants for natural transformation abilities and observed abrogation of this process for all but two mutants, namely, the Δ*pilU* and Δ*comM* mutants (Fig. 3A). The lack of transformation defects in the absence of *comM* was in line with the minor impact observed for similar mutants in diverse V. cholerae strains (62, 63), given that the current transformation assay was based on genomic DNA as transforming material that contained a resistance marker for transformant scoring. In contrast, there was a significant decrease of the transformability in *comM* mutants of V. cholerae and A. baumannii when PCR-amplified fragments served as transforming material, which led to the exchange of single nucleotides or short stretches of DNA (28, 64). PilU, on the other hand, is a secondary retraction ATPase (PilT is the primary enzyme) that is present in many Gram-negative bacteria, including P. aeruginosa, in which it is essential for TFP-mediated twitching motility (65). Related to natural competence, we previously showed that PilU of V. cholerae was dispensable for DNA uptake and transformation (21), which is consistent with our present findings (Fig. 3A). Interestingly, we and others recently demonstrated that V. cholerae’s PilU works solely in conjunction with PilT instead of compensating for the lack of PilT (52, 66), which suggests that it enhances the retraction force instead of behaving as an independent retraction enzyme, as was also suggested for P. aeruginosa (52, 66, 67).

We next established complementation assays to show causality between the lack of pilus or competence genes in this set of knockout strains. Toward this goal, we cloned the genes downstream of the arabinose-inducible P_BAD_ promoter that was located on a miniTn7 transposon (23, 63). These genetically engineered constructs and the parental transposon without any gene inserted downstream of P_BAD_ (TnAraC) were then site-directly integrated into the respective strains (Table 1) and tested for their natural transformability. As shown in Fig. 3B, complete or high levels of complementation were observed under arabinose-inducing conditions for strains lacking *pilT*, *comEA*, *comF*, or *dprA*, compared to those of the WT parental strain and its transposon-carrying derivative (grown in the absence or presence of arabinose). In contrast, complementation of the *pilA*- and *pilQ* deletion strains was less efficient but could be boosted by an increase of the inducer arabinose (Fig. 3C). Collectively, these data confirmed that the transformation defects were caused by the gene-specific deletions and not secondary defects that might have occurred during strain engineering.

### Pilus mutants are impaired in their surface motility.

Recent studies are reporting considerable variations across A. baumannii strains that might also impact and explain their different surface mobilities. For example, considerable variations in amino acid sequences and glycosylation patterns of PilA proteins have been observed (strain A118 lacks the *O*-glycosylated C-terminal serine residue that is conserved in several other strains) (68–70). Moreover, X-ray crystallography highlighted another key difference between A. baumannii PilA proteins, namely, surface electrostatics, which could determine whether pili repulse or adhere to one another (70). Electrostatic adherence could trigger bundle formation, which would influence the strain’s preference for biofilm formation over surface-dependent twitching motility. Given the correlation between TFP biosynthesis and natural transformation of strain A118 (Fig. 1), we queried whether deletion of the pilus and core competence genes would affect such surface-dependent movement. As shown in Fig. 3D, we observed large variations in and between independent experiments, which could again be attributed to the species’ phase variability that is known to affect surface motility (59). Nonetheless, motile versus nonmotile strains were easily differentiable. We demonstrated that all *pil* mutants abrogated surface motility, while deletion of nonpilus competence genes did not impair surface movement. This finding contrasts previous reports by Wilharm et al., who showed that inactivation of both *pilT* and *comEC* abolished transformation and twitching-like motility in A. baumannii strains 07-095 and 07-102 (14). Interestingly, the *pilU* mutant had significantly impaired motility (Fig. 3D). This discrepancy between the *pilU* mutant’s efficient transformability (Fig. 3A) and its inefficient surface motility (Fig. 3D) can be explained by the difference in requisite retraction force. While DNA uptake is unlikely to majorly constrain pilus retraction, the friction between the bacterial cell and the surface material likely requires enhanced force generation by the retraction motor PilT, which is accomplished through recruitment of and assistance by PilU. The absence of surface motility of the *pilU* mutant observed here for A. baumannii phenocopies that of P. aeruginosa (65). Despite this commonality between the two organisms, a recent study by Nolan et al. demonstrated that TFP were dispensable for low levels of natural transformation of P. aeruginosa (29), which contrasted with our findings. Here, we demonstrated that apart from PilU, the pilus components were essential for A. baumannii’s natural transformability (Fig. 3A).

### TFP regulators impact transformation in A. baumannii.

**FIG 4:**
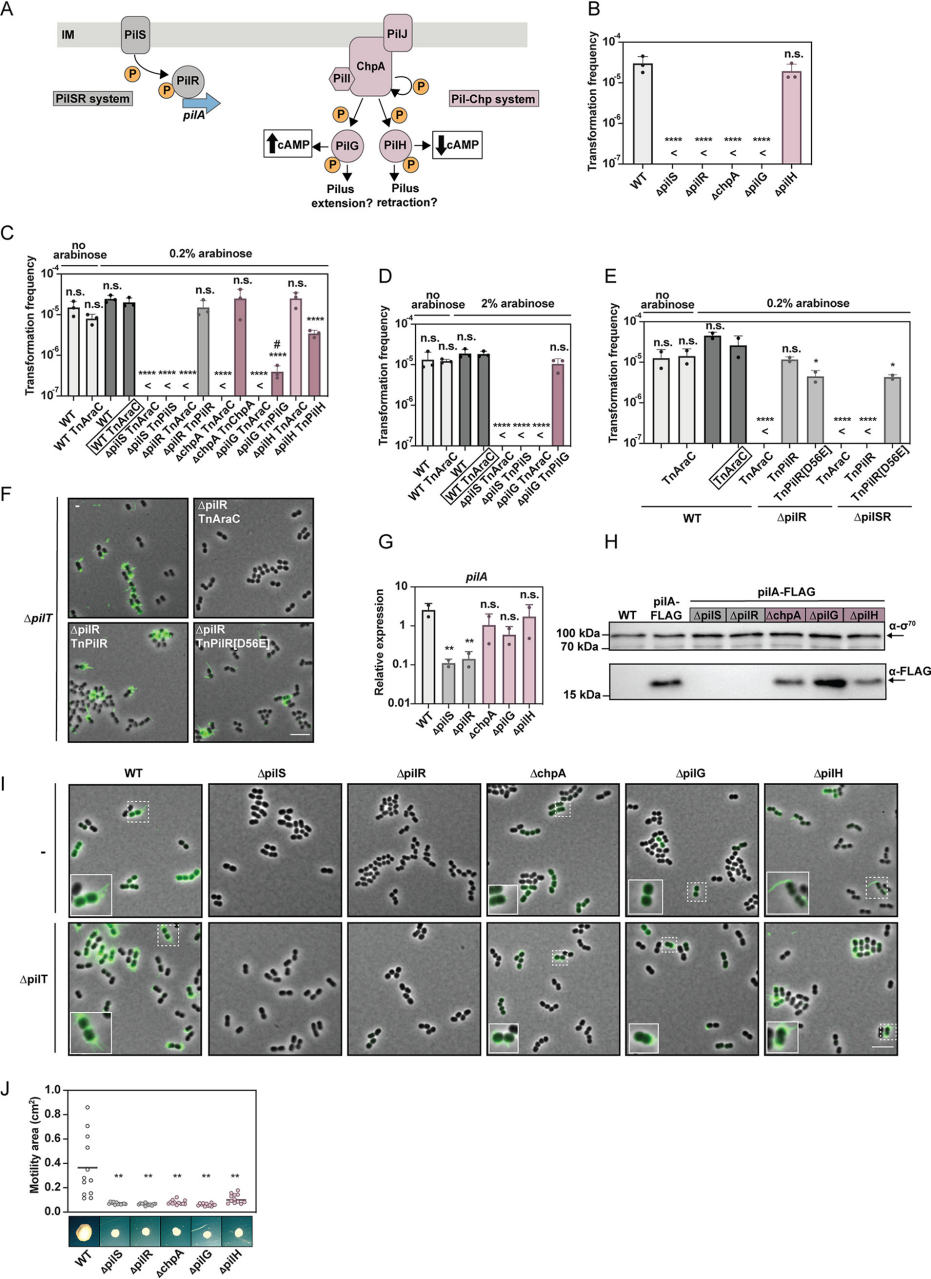
The A. baumannii PilSR and Pil-Chp systems are required for natural transformation and surface motility. (A) Schematic representation of the PilSR and Pil-Chp systems. Left (gray): upon activation, PilS phosphorylates PilR, which promotes expression of *pilA.* Right (purple): PilJ promotes autophosphorylation of ChpA, which subsequently phosphorylates PilG and/or PilH, resulting in increased or decreased cAMP levels, respectively. PilG and PilH were also proposed to foster T4P extension or retraction. (B to E) Transformability of TFP regulation mutants without or with complementing constructs ± inducer, as indicated. Details as in Fig. 3. The WT and WT-TnAraC served as controls. Transformation frequencies are shown as mean value (±SD) from three independent experiments. <, below detection limit (DL). For statistical analyses, a one-way ANOVA with Sidak’s multiple comparisons test was performed on log-transformed data and the different strains’ values were compared to the WT (B) or to the most appropriate control strain (boxed name in panels C to E). #, under detection limit in at least one replicate. *, *P* < 0.05; ****, *P* < 0.0001; n.s. = not significant. (F and I) Imaging of TFP in the regulatory mutants. PilA(A61C) pilus imaging of *pilT*-positive or *pilT*-negative strains (as indicated). (F) The strains were *pilR-*positive or *pilR*-negative and carried complementing *pilR* or its phosphomimetic *pilR*(D56E) variant on a transposon, as indicated. (I) The strains were deleted for the regulatory gene that is indicated above each column. Details as in Fig. 2, with the exception that only the merged images are shown. Bar = 5 μm (enlarged images are 2× magnified). (G) Relative expression of *pilA* in the regulatory mutants. Average values (±SD) from two independent experiments are shown, and statistics reflect a two-way ANOVA with Tukey’s multiple comparisons test in which each strain was compared to the WT. **, *P* < 0.01; n.s., not significant. (H) Detection of PilA-FLAG in the different regulatory mutants. Representative images of two independent replicates. Details as in Fig. 1. (J) TFP regulatory mutants are nonmotile. The surface motility of the PilSR/ChpA system mutants is shown. Details as in Fig. 3D. The motility values of each strain were compared to those of the WT using Brown-Forsythe and Welch ANOVA tests with unpaired *t* test with Welch’s correction. **, *P* < 0.01.

Given the parallels and discrepancies between P. aeruginosa and A. baumannii, we next considered the impact of pilus-specific regulators on surface-dependent motility and transformation of A. baumannii. We were especially interested in the PilSR TCS and the Pil-Chp chemosensory systems (Fig. 4A), as homologs for these regulatory proteins exist in A. baumannii (Table 3). Notably, Leong et al. recently showed that, while important for twitching motility, these signal transduction-related systems (e.g., PilS, PilR, and PilG) were fully dispensable for natural transformation in A. baylyi (strains ADP1 and BD413) (48), which was recently confirmed for the Pil-Chp system (53). Given the differences we found in natural transformability and TFP production between A. baumannii and A. baylyi, we were interested in these systems in A. baumannii. In contrast to A. baylyi, deletion of *pilS* or *pilR* as well as *chpA* or *pilG* in A. baumannii completely abolished transformation, while the absence of *pilH* did not change the strain’s transformation efficiency (Fig. 4B). Complementation restored natural transformability in the mutants at regular (Fig. 4C) or elevated induction levels (Fig. 4D), except for the *pilS* deletion strain, which could not be complemented under the tested conditions. However, when we tested a *pilSR* double mutant, we restored its function through production of a phosphomimetic PilR variant (D56E), while the native and unphosphorylated PilR was insufficient for restoring transformation in the mutant (Fig. 4E). In contrast, both variants efficiently complemented a *pilR* single knockout in which the PilS protein was maintained to restore piliation (Fig. 4F) and natural transformation (Fig. 4E). Therefore, these data confirm that PilS is required for natural transformation in A. baumannii due to its phosphotransfer to PilR. Moreover, these data also suggest that the observed TFP production heterogeneity is not based on the level of phosphorylated PilR given that the phosphomimetic version should be expressed from the P_BAD_ promoter in the whole population. In addition, and similar to the situation for the WT (Fig. 1B), no transformants were detected for the complementing PilR(D56E) variant for samples taken after 240 min of growth (*n* = 2), suggesting that an absence of phosphorylated PilR is not the reason for the lack of transformation at this later time point during growth.

Given the strong transformation phenotype of most of the regulatory mutants, we next tested their *pilA* transcript and PilA protein levels. For the *pilS* and *pilR* mutants, *pilA* expression levels were significantly reduced (Fig. 4G), and tagged PilA proteins were undetectable (Fig. 4H). On the contrary, the absence of *chpA*, *pilG*, or *pilH* significantly impacted neither the *pilA* transcript levels nor the PilA protein levels (Fig. 4G and H). We therefore speculated that the pilus assembly/disassembly might be affected in the *chpA* and *pilG* mutants, which could explain their nontransformability (Fig. 4B). Hence, we combined the cysteine knock-in *pilA* allele with the various gene deletions and imaged potential pili in otherwise WT or *pilT*-minus background strains. Consistent with the absence of the major pilins in the Western blot analysis (Fig. 4H), neither pili nor the retracted inner membrane-localized pilin subunit were detectable in the *pilS* and *pilR* mutants (Fig. 4I). Extended pili were also undetectable in the absence of ChpA and PilG. This is consistent with previous work in P. aeruginosa that showed production of the major pilin but no surface-exposed and therefore shearable pili when similar mutants were investigated. Nonetheless, in our experiments, fluorescent puncta were observed close to the cell surface, which suggests that these strains might initiate pilus elongation but are unable to assemble full-length pili. This phenotype was partially reversed when the retraction enzyme PilT was absent, in which case extended pili/pilus bundles were visible (Fig. 4I). The labeling of the cell bodies of the *chpA* and *pilG* mutants by the thiol-reactive maleimide dye supports our idea that the initiation of pilus assembly takes place but that the very short pili seen as puncta in our images are quickly retracted to transport the dye-labeled pilins back into the inner membrane. This phenotype might be caused by an imbalanced PilH protein, which is known to enhance the function of PilT in P. aeruginosa (37). These data are also in line with a previous observation that *chpA* and *pilG* mutants of P. aeruginosa remained susceptible to pili-specific phage infections, which was not the case for an extension-defective *pilB* mutant (37). The *pilH* mutant, on the other hand, had surface-exposed long pili but contained fewer bacteria with labeled cell bodies than the WT conditions (Fig. 4I). We therefore hypothesized that pilus elongation is favored over retraction through an imbalanced PilG phosphorylation in this mutant.

Lastly, we tested the regulatory mutants for their ability to move on solid surfaces. As shown in Fig. 4J, all mutants of the two systems showed strong motility defects relative to the WT. Interestingly, we observed that the *pilH* mutant exhibited strongly impaired surface motility despite being piliated and fully transformable. This discrepancy between fully functional transformation but a lack of proficient motility phenocopies the *pilU* mutant. These results suggest that PilH might play a role in the recruitment or activation of PilU, potentially to enhance high-force pilus retraction.

### Conclusion.

In this study, we sought to better understand how the poorly studied human pathogen A. baumannii engages in horizontal gene transfer and, precisely, in natural competence for transformation. We showed that A. baumannii has a tight time window of natural transformability under the tested conditions, which correlated with the bacterium’s TFP production. Consistent with this finding, we demonstrated that this pilus is essential for the pathogen’s natural transformation and that regulatory circuits resembling those of P. aeruginosa play an important part in the production and assembly of the TFP and therefore also natural transformation. Finally, we also supported previous notions that retraction forces can be enhanced by the secondary retraction ATPase PilU, which is dispensable for the DNA uptake process.

Collectively, our data highlight that natural transformation dynamics and pilus regulation in A. baumannii strain A118 vastly differs from those in the model organism A. baylyi, which suggests that common generalizations among these organisms should be taken with caution. Because A. baumannii is a current and urgent threat to human health due to its frequent multidrug resistance, it is important to be able to predict how this pathogen is likely to mutate and the best ways of dealing with infections when it does. Overall, this work sheds important light on mechanisms by which A. baumannii can acquire foreign DNA, and therefore antimicrobial resistance genes, and will assist researchers in better understanding the evolution of A. baumannii as a public health threat.

## MATERIALS AND METHODS

### Bacterial strains, plasmids, and culture conditions.

The bacterial strains and plasmids used in this study are listed in Table 1. Bacterial strains were grown aerobically in lysogeny broth medium (LB; 1% tryptone, 0.5% yeast extract, 1% sodium chloride; Carl Roth, Germany) or on LB agar plates at 37°C. For a selection of Acinetobacter baumannii strains after bi- and triparental mating, CHROMagar Acinetobacter plates were prepared following the manufacturer’s instructions (CHROMagar, France).

For transformation assays, phosphate-buffered LB agar plates (pH 6.0) were used. For induction of the P_BAD_ promoter, l-arabinose was added to the medium at a final concentration of 0.2% or 2%, as indicated.

The following antibiotics were used at these final concentrations whenever required: kanamycin (50 μg/ml), gentamicin (15 μg/ml), and ampicillin (100 μg/ml).

### Long-read whole-genome sequencing (PacBio) and *de novo* assembly.

The isolation of genomic DNA (gDNA) and whole-genome sequencing was performed as previously described (63) with minor modifications. Briefly, A. baumannii strain A118 (ATCC BAA-2093) was back diluted 1:100 from an overnight culture and grown aerobically in 25 ml LB medium for 4 h at 37°C (optical density at 600 nm [OD_600_] 2.5 to 3.0). After 15 ml of this culture was harvested by centrifugation, gDNA was isolated using Qiagen’s Genomic DNA buffer set combined with a 500/G Genomic-tip. The alcohol-precipitated DNA was ultimately dissolved in 10 mM Tris-Cl buffer (pH 8.0). Further sample processing, sequencing, and *de novo* assembly were performed by the Genomic Technology Facility of the University of Lausanne as described (63). The assembled genome was annotated upon submission to the NCBI database using their Prokaryotic Genome Annotation Pipeline (PGAP). The sequencing results are summarized in Table 2.

### Genetic engineering of Acinetobacter baumannii.

A. baumannii mutants or variants were constructed using a standard allelic exchange approach with the counterselectable suicide vectors pGP704-Sac28 or pGP704Sac-kan (71, 72). Derivatives of these plasmids containing deletion constructs of the respective genes or site-directed allele exchanges (of *pilA*) were transferred to A. baumannii through mating with E. coli S17-1λpir. The deletion constructs were based on the PCR amplification of the flanking regions of the desired genetic regions and, when needed, the *aph* cassette (Kan^r^) as the selection marker using PWO polymerase (Roche) or Q5 polymerase (BioLabs). Site-directed changes in the *pilA* allele were inserted using modified primers. The amplified fragments were joined by overlap extension PCR or Golden Gate assembly (73) and were cloned inside the suicide plasmids. The correct cloning products were screened for by colony PCR of the E. coli transformants (with GoTaq polymerase; Promega) and ultimately confirmed by Sanger sequencing (Microsynth, Switzerland).

Construction of inducible genes-of-interest was accomplished by placing the respective gene under the control of the arabinose-inducible promoter P_BAD_ inside the miniTn7 transposon TnAraC (23, 63). The resulting transposons were transferred to the respective A. baumannii strains through a standard triparental mating approach (74).

### Natural transformation assay.

The transformation protocol used in this study was adapted from Harding et al. (15). Briefly, the bacteria were grown overnight in LB medium, back diluted 1:100 into 2 ml of LB medium with or without 0.2% or 2% l-arabinose, and further cultured aerobically until they reached an OD_600_ of approximately 0.65 (or, for the time course experiment, the OD_600_ value indicated in Fig. 1). Next, 20 μl of the bacterial culture was mixed with 1 μg of isolated genomic DNA of strain A118Δ*hcp1::kan*, and 20 μl of this mixture was spotted onto pH-buffered LB agar plates (pH 6; without or with 0.2% or 2% arabinose). The plates were incubated for 2 h at 37°C. Following this incubation period, the cells were scraped off from the plate with a sterile loop and resuspended in 200 μl of LB medium. Serial dilutions were spotted in duplicate on kanamycin-containing LB agar plates to select the transformants and on plain LB agar plates to assess the total number of CFU. Transformation frequency was calculated as the CFU number of transformants divided by the total number of bacteria. Averages of at least two biologically independent experiments are provided. Transformation frequencies were log-transformed (75) and statistically significant differences were determined as described in the figure legends. When no transformants were recovered, the transformation frequencies were set to the detection limit to allow the calculation of the average value of all biologically independent replicates and for statistical analyses using the program Prism (GraphPad software; San Diego, USA).

### TFP-dependent surface motility assay.

The bacterial strains were streaked on LB agar plates and incubated at 37°C overnight to obtain single colonies. Bacterial material was retrieved from a single colony with a toothpick and gently applied onto a freshly prepared 1% agar-only plate. The plates were sealed with parafilm and incubated at 37°C for 4 days. Following the incubation, pictures of the plates were taken with an M80 Stereo Zoom microscope equipped with an MC 170 HD camera (both from Leica). The motility area was determined using ImageJ software (imagej.nih.gov/ij/). Results of four biological replicates consisting of three technical replicates for each strain are provided. Significant differences were determined using Prism, as indicated in the legends to Fig. 1 and 4.

### Gene expression determination using quantitative reverse transcription-PCR (qRT-PCR).

Back-diluted cultures were grown at 37°C in several parallel 2 ml LB medium-containing tubes for the indicated amount of time (for the time course experiment) or up to an OD_600_ of 0.65. The bacterial cells (1.5 ml to 8 ml, depending on the growth phase) were harvested by centrifugation for 3 min at 4°C. The cell pellets were washed once with ice-cold PBS, resuspended in TRI reagent (Sigma-Aldrich), vortexed for a few seconds, and then flash-frozen on dry ice before their storage at –80°C. The RNA extraction, DNase treatment, reverse transcription, and quantitative PCR (qPCR) were performed as previously described (76). Relative gene expression values were calculated based on normalization against the transcript levels of the housekeeping gene *gyrA*. Averages of at least three independent biological experiments are shown. Data were log-transformed (75) and statistical differences were assessed using the Prism software. Details on the statistical analyses are provided in the figure legends to Fig. 1 and 4.

### SDS-PAGE and Western blotting.

To verify the production of PilA-FLAG proteins, cell lysates were prepared as described previously with minor modifications (77). Briefly, after overnight growth, the bacterial culture was back diluted 1:100 in 2 ml of LB and grown at 37°C for the indicated amount of time (for time course experiments) or up to an OD_600_ of ∼0.65. At the time of harvesting, the bacteria were centrifuged for 3 min, and the pelleted cells were resuspended in 2× Laemmli buffer, whereby the volume was adjusted according to the total number of bacteria (100 μl buffer per OD_600_ unit). The resuspended samples were incubated at 95°C for 15 min. Proteins were separated by SDS-PAGE using 15% resolving gels and blotted onto\polyvinylidene difluoride (PVDF) membranes as previously described (76). Primary monoclonal antibodies against the FLAG tag (ANTI-FLAG M2; Sigma-Aldrich) were used at 1:2,000 dilution, and goat anti-mouse antibody—horseradish peroxidase (HRP) served as the secondary antibody (diluted 1:5,000; Sigma-Aldrich). Sigma70 was detected as a loading control using Direct-Blot anti-E. coli Sigma70-HRP-conjugated antibodies at a dilution of 1:10,000 (BioLegend, USA distributed via Brunschwig, Switzerland). Lumi-Light^PLUS^ Western blotting substrate (Roche) served as the HRP substrate. Luminescent signals were detected using a ChemiDoc XRS+ station (Bio-Rad).

### Microscopy.

Bacteria were grown until an OD_600_ of ∼0.65 and then spotted onto thin agarose pads that were mounted onto glass slides (1.2% agarose dissolved in 0.5× PBS). The cells were illuminated with an HXP120 lamp and imaged through a Plan-Apochromat 100×/numerical aperture 1.4 Ph3 oil objective by an AxioCam MRm camera attached to an Axio Imager M2 epi-fluorescence microscope (Zeiss). The Zeiss software ZEN 2.6 and ImageJ (imagej.nih.gov/ij/) were used for image acquisition and analysis, respectively.

### Type IV pilus labeling.

Pilus labeling was performed as previously described with minor modifications (23, 50). Briefly, control strains or genetically modified bacteria with modified *pilA* alleles (encoding site-specific amino acid changes to knock-in a new cysteine residue) were pregrown overnight at 37°C, back diluted 1:100 in 2 ml LB (supplemented without or with l-arabinose, as indicated), and grown at 37°C until they reached an OD_600_ of approximately 0.65. Alexa Fluor 488 C5 maleimide (AF-488-Mal; Thermo Fisher Scientific) was added to 100 μl of the bacterial culture at a final concentration of 25 μg/ml, gently mixed, and incubated for 15 min at room temperature in the dark. Cells were subsequently harvested by centrifugation (5,000 × *g* for 1 min.), washed once in 1× PBS, and resuspended in 30 μl of PBS before being imaged as described above.

### Data availability.

The assembled genome sequence and all raw data were deposited into NCBI under GenBank accession number CP059039, BioSample number SAMN15507634, and the Sequence Read Archive (SRA) accession number SRX8711359.

## References

[1] Rice LB. 2008. Federal funding for the study of antimicrobial resistance in nosocomial pathogens: no ESKAPE. J Infect Dis 197:1079–1081. doi:10.1086/533452. 18419525

[2] Munoz-Price LS, Weinstein RA. 2008. *Acinetobacter* infection. N Engl J Med 358:1271–1281. doi:10.1056/NEJMra070741. 18354105

[3] Touchon M, Cury J, Yoon EJ, Krizova L, Cerqueira GC, Murphy C, Feldgarden M, Wortman J, Clermont D, Lambert T, Grillot-Courvalin C, Nemec A, Courvalin P, Rocha EP. 2014. The genomic diversification of the whole *Acinetobacter* genus: origins, mechanisms, and consequences. Genome Biol Evol 6:2866–2882. doi:10.1093/gbe/evu225. 25313016 PMC4224351

[4] Lorenz MG, Wackernagel W. 1994. Bacterial gene transfer by natural genetic transformation in the environment. Microbiol Rev 58:563–602. 7968924 10.1128/mr.58.3.563-602.1994 PMC372978

[5] Johnston C, Martin B, Fichant G, Polard P, Claverys JP. 2014. Bacterial transformation: distribution, shared mechanisms and divergent control. Nat Rev Microbiol 12:181–196. doi:10.1038/nrmicro3199. 24509783

[6] Seitz P, Blokesch M. 2013. Cues and regulatory pathways involved in natural competence and transformation in pathogenic and environmental Gram-negative bacteria. FEMS Microbiol Rev 37:336–363. doi:10.1111/j.1574-6976.2012.00353.x. 22928673

[7] Dubnau D, Blokesch M. 2019. Mechanisms of DNA uptake by naturally competent bacteria. Annu Rev Genet 53:217–237. doi:10.1146/annurev-genet-112618-043641. 31433955

[8] Cooper RM, Tsimring L, Hasty J. 2017. Inter-species population dynamics enhance microbial horizontal gene transfer and spread of antibiotic resistance. Elife 6:e25950. doi:10.7554/eLife.25950. 29091031 PMC5701796

[9] Lin L, Ringel PD, Vettiger A, Durr L, Basler M. 2019. DNA uptake upon T6SS-dependent prey cell lysis induces SOS response and reduces fitness of *Acinetobacter baylyi*. Cell Rep 29:1633–1644.e4. doi:10.1016/j.celrep.2019.09.083. 31693901

[10] Traglia GM, Quinn B, Schramm ST, Soler Bistue A, Ramirez MS. 2016. Serum albumin and Ca^2+^ are natural competence inducers in the human pathogen *Acinetobacter baumannii*. Antimicrob Agents Chemother 60:4920–4929. doi:10.1128/AAC.00529-16. 27270286 PMC4958237

[11] Quinn B, Traglia GM, Nguyen M, Martinez J, Liu C, Fernandez JS, Ramirez MS. 2019. Effect of host human products on natural transformation in *Acinetobacter baumannii*. Curr Microbiol 76:950–953. doi:10.1007/s00284-017-1417-5. 29332139 PMC6890376

[12] Godeux AS, Lupo A, Haenni M, Guette-Marquet S, Wilharm G, Laaberki MH, Charpentier X. 2018. Fluorescence-based detection of natural transformation in drug-resistant *Acinetobacter baumannii*. J Bacteriol 200:e00181-18. doi:10.1128/JB.00181-18. 30012729 PMC6148472

[13] Ramirez MS, Don M, Merkier AK, Bistue AJ, Zorreguieta A, Centron D, Tolmasky ME. 2010. Naturally competent *Acinetobacter baumannii* clinical isolate as a convenient model for genetic studies. J Clin Microbiol 48:1488–1490. doi:10.1128/JCM.01264-09. 20181905 PMC2849597

[14] Wilharm G, Piesker J, Laue M, Skiebe E. 2013. DNA uptake by the nosocomial pathogen *Acinetobacter baumannii* occurs during movement along wet surfaces. J Bacteriol 195:4146–4153. doi:10.1128/JB.00754-13. 23852865 PMC3754747

[15] Harding CM, Tracy EN, Carruthers MD, Rather PN, Actis LA, Munson RS. Jr., 2013. *Acinetobacter baumannii* strain M2 produces type IV pili which play a role in natural transformation and twitching motility but not surface-associated motility. mBio 4:e00360-13. doi:10.1128/mBio.00360-13. 23919995 PMC3735195

[16] Wilharm G, Skiebe E. 2019. Methods for natural transformation in *Acinetobacter baumannii*. Methods Mol Biol 1946:75–85. doi:10.1007/978-1-4939-9118-1_8. 30798546

[17] Carruthers MD, Harding CM, Baker BD, Bonomo RA, Hujer KM, Rather PN, Munson RS. Jr., 2013. Draft genome sequence of the clinical isolate *Acinetobacter nosocomialis* strain M2. Genome Announc 1:e00906-13. 24201195 10.1128/genomeA.00906-13 PMC3820776

[18] Wilharm G, Skiebe E, Higgins PG, Poppel MT, Blaschke U, Leser S, Heider C, Heindorf M, Brauner P, Jäckel U, Böhland K, Cuny C, Łopińska A, Kaminski P, Kasprzak M, Bochenski M, Ciebiera O, Tobółka M, Żołnierowicz KM, Siekiera J, Seifert H, Gagné S, Salcedo SP, Kaatz M, Layer F, Bender JK, Fuchs S, Semmler T, Pfeifer Y, Jerzak L. 2017. Relatedness of wildlife and livestock avian isolates of the nosocomial pathogen *Acinetobacter baumannii* to lineages spread in hospitals worldwide. Environ Microbiol 19:4349–4364. doi:10.1111/1462-2920.13931. 28925528

[19] Hu Y, He L, Tao X, Meng F, Zhang J. 2019. High DNA uptake capacity of international clone II *Acinetobacter baumannii* detected by a novel planktonic natural transformation assay. Front Microbiol 10:2165. doi:10.3389/fmicb.2019.02165. 31616393 PMC6768954

[20] Meibom KL, Blokesch M, Dolganov NA, Wu C-Y, Schoolnik GK. 2005. Chitin induces natural competence in *Vibrio cholerae*. Science 310:1824–1827. doi:10.1126/science.1120096. 16357262

[21] Seitz P, Blokesch M. 2013. DNA-uptake machinery of naturally competent *Vibrio cholerae*. Proc Natl Acad Sci U S A 110:17987–17992. doi:10.1073/pnas.1315647110. 24127573 PMC3816411

[22] Seitz P, Pezeshgi Modarres H, Borgeaud S, Bulushev RD, Steinbock LJ, Radenovic A, Dal Peraro M, Blokesch M. 2014. ComEA is essential for the transfer of external DNA into the periplasm in naturally transformable *Vibrio cholerae* cells. PLoS Genet 10:e1004066. doi:10.1371/journal.pgen.1004066. 24391524 PMC3879209

[23] Adams DW, Stutzmann S, Stoudmann C, Blokesch M. 2019. DNA-uptake pili of *Vibrio cholerae* are required for chitin colonization and capable of kin recognition via sequence-specific self-interaction. Nat Microbiol 4:1545–1557. doi:10.1038/s41564-019-0479-5. 31182799 PMC6708440

[24] Ellison CK, Dalia TN, Vidal Ceballos A, Wang JC, Biais N, Brun YV, Dalia AB. 2018. Retraction of DNA-bound type IV competence pili initiates DNA uptake during natural transformation in *Vibrio cholerae*. Nat Microbiol 3:773–780. doi:10.1038/s41564-018-0174-y. 29891864 PMC6582970

[25] Craig L, Forest KT, Maier B. 2019. Type IV pili: dynamics, biophysics and functional consequences. Nat Rev Microbiol 17:429–440. doi:10.1038/s41579-019-0195-4. 30988511

[26] Seitz P, Blokesch M. 2014. DNA transport across the outer and inner membranes of naturally transformable *Vibrio cholerae* is spatially but not temporally coupled. mBio 5:e01409-14. doi:10.1128/mBio.01409-14. 25139903 PMC4147865

[27] Marie L, Rapisarda C, Morales V, Berge M, Perry T, Soulet AL, Gruget C, Remaut H, Fronzes R, Polard P. 2017. Bacterial RadA is a DnaB-type helicase interacting with RecA to promote bidirectional D-loop extension. Nat Commun 8:15638. doi:10.1038/ncomms15638. 28561029 PMC5512693

[28] Nero TM, Dalia TN, Wang JC, Kysela DT, Bochman ML, Dalia AB. 2018. ComM is a hexameric helicase that promotes branch migration during natural transformation in diverse Gram-negative species. Nucleic Acids Res 46:6099–6111. doi:10.1093/nar/gky343. 29722872 PMC6158740

[29] Nolan LM, Turnbull L, Katrib M, Osvath SR, Losa D, Lazenby JJ, Whitchurch CB. 2020. *Pseudomonas aeruginosa* is capable of natural transformation in biofilms. Microbiology (Reading) 166:995–1003. doi:10.1099/mic.0.000956. 32749953 PMC7660920

[30] Ishimoto KS, Lory S. 1992. Identification of *pilR*, which encodes a transcriptional activator of the *Pseudomonas aeruginosa* pilin gene. J Bacteriol 174:3514–3521. doi:10.1128/jb.174.11.3514-3521.1992. 1317379 PMC206036

[31] Boyd JM, Koga T, Lory S. 1994. Identification and characterization of PilS, an essential regulator of pilin expression in *Pseudomonas aeruginosa*. Mol Gen Genet 243:565–574. doi:10.1007/BF00284205. 7911557

[32] Hobbs M, Collie ES, Free PD, Livingston SP, Mattick JS. 1993. PilS and PilR, a two-component transcriptional regulatory system controlling expression of type 4 fimbriae in *Pseudomonas aeruginosa*. Mol Microbiol 7:669–682. doi:10.1111/j.1365-2958.1993.tb01158.x. 8097014

[33] Kilmury SL, Burrows LL. 2016. Type IV pilins regulate their own expression via direct intramembrane interactions with the sensor kinase PilS. Proc Natl Acad Sci U S A 113:6017–6022. doi:10.1073/pnas.1512947113. 27162347 PMC4889343

[34] Kilmury SLN, Burrows LL. 2018. The *Pseudomonas aeruginosa* PilSR two-component system regulates both twitching and swimming motilities. mBio 9:e01310-18. doi:10.1128/mBio.01310-18. 30042200 PMC6058289

[35] Whitchurch CB, Leech AJ, Young MD, Kennedy D, Sargent JL, Bertrand JJ, Semmler AB, Mellick AS, Martin PR, Alm RA, Hobbs M, Beatson SA, Huang B, Nguyen L, Commolli JC, Engel JN, Darzins A, Mattick JS. 2004. Characterization of a complex chemosensory signal transduction system which controls twitching motility in *Pseudomonas aeruginosa*. Mol Microbiol 52:873–893. doi:10.1111/j.1365-2958.2004.04026.x. 15101991

[36] Graham KJ, Burrows LL. 2020. More than a feeling: microscopy approaches to understanding surface-sensing mechanisms. J Bacteriol doi:10.1128/JB.00492-20. PMC8095462 33077631

[37] Bertrand JJ, West JT, Engel JN. 2010. Genetic analysis of the regulation of type IV pilus function by the Chp chemosensory system of *Pseudomonas aeruginosa*. J Bacteriol 192:994–1010. doi:10.1128/JB.01390-09. 20008072 PMC2812951

[38] Inclan YF, Persat A, Greninger A, Von Dollen J, Johnson J, Krogan N, Gitai Z, Engel JN. 2016. A scaffold protein connects type IV pili with the Chp chemosensory system to mediate activation of virulence signaling in *Pseudomonas aeruginosa*. Mol Microbiol 101:590–605. doi:10.1111/mmi.13410. 27145134 PMC4980298

[39] Persat A, Inclan YF, Engel JN, Stone HA, Gitai Z. 2015. Type IV pili mechanochemically regulate virulence factors in *Pseudomonas aeruginosa*. Proc Natl Acad Sci U S A 112:7563–7568. doi:10.1073/pnas.1502025112. 26041805 PMC4475988

[40] Darzins A. 1994. Characterization of a *Pseudomonas aeruginosa* gene cluster involved in pilus biosynthesis and twitching motility: sequence similarity to the chemotaxis proteins of enterics and the gliding bacterium *Myxococcus xanthus*. Mol Microbiol 11:137–153. doi:10.1111/j.1365-2958.1994.tb00296.x. 7908398

[41] Fulcher NB, Holliday PM, Klem E, Cann MJ, Wolfgang MC. 2010. The *Pseudomonas aeruginosa* Chp chemosensory system regulates intracellular cAMP levels by modulating adenylate cyclase activity. Mol Microbiol 76:889–904. doi:10.1111/j.1365-2958.2010.07135.x. 20345659 PMC2906755

[42] Silversmith RE, Wang B, Fulcher NB, Wolfgang MC, Bourret RB. 2016. Phosphoryl group flow within the *Pseudomonas aeruginosa* Pil-Chp chemosensory system: differential function of the eight phosphotransferase and three receiver domains. J Biol Chem 291:17677–17691. doi:10.1074/jbc.M116.737528. 27354279 PMC5016163

[43] Wolfgang MC, Lee VT, Gilmore ME, Lory S. 2003. Coordinate regulation of bacterial virulence genes by a novel adenylate cyclase-dependent signaling pathway. Dev Cell 4:253–263. doi:10.1016/s1534-5807(03)00019-4. 12586068

[44] Juni E, Janik A. 1969. Transformation of *Acinetobacter calco-aceticus* (Bacterium anitratum). J Bacteriol 98:281–288. doi:10.1128/JB.98.1.281-288.1969. 5781579 PMC249934

[45] Cruze JA, Singer JT, Finnerty WR. 1979. Conditions for quantitative transformation in *Acinetobacter calcoaceticus*. Curr Microbiol 3:129–132. doi:10.1007/BF02601853.

[46] Porstendörfer D, Gohl O, Mayer F, Averhoff B. 2000. ComP, a pilin-like protein essential for natural competence in *Acinetobacter* sp. Strain BD413: regulation, modification, and cellular localization. J Bacteriol 182:3673–3680. doi:10.1128/jb.182.13.3673-3680.2000. 10850981 PMC94537

[47] Ramirez MS, Adams MD, Bonomo RA, Centron D, Tolmasky ME. 2011. Genomic analysis of *Acinetobacter baumannii* A118 by comparison of optical maps: identification of structures related to its susceptibility phenotype. Antimicrob Agents Chemother 55:1520–1526. doi:10.1128/AAC.01595-10. 21282446 PMC3067174

[48] Leong CG, Bloomfield RA, Boyd CA, Dornbusch AJ, Lieber L, Liu F, Owen A, Slay E, Lang KM, Lostroh CP. 2017. The role of core and accessory type IV pilus genes in natural transformation and twitching motility in the bacterium *Acinetobacter baylyi*. PLoS One 12:e0182139. doi:10.1371/journal.pone.0182139. 28771515 PMC5542475

[49] Blair KM, Turner L, Winkelman JT, Berg HC, Kearns DB. 2008. A molecular clutch disables flagella in the *Bacillus subtilis* biofilm. Science 320:1636–1638. doi:10.1126/science.1157877. 18566286

[50] Ellison CK, Kan J, Dillard RS, Kysela DT, Ducret A, Berne C, Hampton CM, Ke Z, Wright ER, Biais N, Dalia AB, Brun YV. 2017. Obstruction of pilus retraction stimulates bacterial surface sensing. Science 358:535–538. doi:10.1126/science.aan5706. 29074778 PMC5805138

[51] Kelley LA, Mezulis S, Yates CM, Wass MN, Sternberg MJ. 2015. The Phyre2 web portal for protein modeling, prediction and analysis. Nat Protoc 10:845–858. doi:10.1038/nprot.2015.053. 25950237 PMC5298202

[52] Adams DW, Pereira JM, Stoudmann C, Stutzmann S, Blokesch M. 2019. The type IV pilus protein PilU functions as a PilT-dependent retraction ATPase. PLoS Genet 15:e1008393. doi:10.1371/journal.pgen.1008393. 31525185 PMC6762196

[53] Ellison CK, Dalia TN, Shaevitz JW, Gitai Z, Dalia AB. 2020. Novel mechanisms of type IV pilus regulation in *Acinetobacter baylyi*, version 2. bioRxiv https://www.biorxiv.org/content/10.1101/2020.09.28.317149v2.

[54] Maamar H, Dubnau D. 2005. Bistability in the *Bacillus subtilis* K-state (competence) system requires a positive feedback loop. Mol Microbiol 56:615–624. doi:10.1111/j.1365-2958.2005.04592.x. 15819619 PMC3831615

[55] Smits WK, Eschevins CC, Susanna KA, Bron S, Kuipers OP, Hamoen LW. 2005. Stripping *Bacillus*: ComK auto-stimulation is responsible for the bistable response in competence development. Mol Microbiol 56:604–614. doi:10.1111/j.1365-2958.2005.04488.x. 15819618

[56] Maamar H, Raj A, Dubnau D. 2007. Noise in gene expression determines cell fate in *Bacillus subtilis*. Science 317:526–529. doi:10.1126/science.1140818. 17569828 PMC3828679

[57] Singh JK, Adams FG, Brown MH. 2018. Diversity and function of capsular polysaccharide in *Acinetobacter baumannii*. Front Microbiol 9:3301. doi:10.3389/fmicb.2018.03301. 30687280 PMC6333632

[58] Wyres KL, Cahill SM, Holt KE, Hall RM, Kenyon JJ. 2020. Identification of *Acinetobacter baumannii* loci for capsular polysaccharide (KL) and lipooligosaccharide outer core (OCL) synthesis in genome assemblies using curated reference databases compatible with Kaptive. Microb Genom 6:e000339. doi:10.1099/mgen.0.000339. PMC7200062 32118530

[59] Tipton KA, Dimitrova D, Rather PN. 2015. Phase-variable control of multiple phenotypes in *Acinetobacter baumannii* strain AB5075. J Bacteriol 197:2593–2599. doi:10.1128/JB.00188-15. 26013481 PMC4518826

[60] Tucker AT, Nowicki EM, Boll JM, Knauf GA, Burdis NC, Trent MS, Davies BW. 2014. Defining gene-phenotype relationships in *Acinetobacter baumannii* through one-step chromosomal gene inactivation. mBio 5:e01313-14. doi:10.1128/mBio.01313-14. 25096877 PMC4128354

[61] Matthey N, Blokesch M. 2016. The DNA-uptake process of naturally competent *Vibrio cholerae*. Trends Microbiol 24:98–110. doi:10.1016/j.tim.2015.10.008. 26614677

[62] Jaskólska M, Stutzmann S, Stoudmann C, Blokesch M. 2018. QstR-dependent regulation of natural competence and type VI secretion in *Vibrio cholerae*. Nucleic Acids Res 46:10619–10634. doi:10.1093/nar/gky717. 30102403 PMC6237807

[63] Stutzmann S, Blokesch M. 2020. Comparison of chitin-induced natural transformation in pandemic *Vibrio cholerae* O1 El Tor strains. Environ Microbiol 22:4149–4166. doi:10.1111/1462-2920.15214. 32860313 PMC7693049

[64] Godeux AS, Svedholm E, Lupo A, Haenni M, Venner S, Laaberki MH, Charpentier X. 2020. Scarless removal of large resistance island AbaR results in antibiotic susceptibility and increased natural transformability in *Acinetobacter baumannii*. Antimicrob Agents Chemother 64:e00951-20. doi:10.1128/AAC.00951-20. 32778544 PMC7508600

[65] Chiang P, Sampaleanu LM, Ayers M, Pahuta M, Howell PL, Burrows LL. 2008. Functional role of conserved residues in the characteristic secretion NTPase motifs of the Pseudomonas aeruginosa type IV pilus motor proteins PilB, PilT and PilU. Microbiology (Reading) 154:114–126. doi:10.1099/mic.0.2007/011320-0. 18174131

[66] Chlebek JL, Hughes HQ, Ratkiewicz AS, Rayyan R, Wang JC, Herrin BE, Dalia TN, Biais N, Dalia AB. 2019. PilT and PilU are homohexameric ATPases that coordinate to retract type IVa pili. PLoS Genet 15:e1008448. doi:10.1371/journal.pgen.1008448. 31626631 PMC6821130

[67] Tala L, Fineberg A, Kukura P, Persat A. 2019. *Pseudomonas aeruginosa* orchestrates twitching motility by sequential control of type IV pili movements. Nat Microbiol 4:774–780. doi:10.1038/s41564-019-0378-9 30804544 PMC6522360

[68] Harding CM, Nasr MA, Kinsella RL, Scott NE, Foster LJ, Weber BS, Fiester SE, Actis LA, Tracy EN, Munson RS, Jr., Feldman MF. 2015. *Acinetobacter* strains carry two functional oligosaccharyltransferases, one devoted exclusively to type IV pilin, and the other one dedicated to O-glycosylation of multiple proteins. Mol Microbiol 96:1023–1041. doi:10.1111/mmi.12986. 25727908

[69] Piepenbrink KH, Lillehoj E, Harding CM, Labonte JW, Zuo X, Rapp CA, Munson RS, Jr., Goldblum SE, Feldman MF, Gray JJ, Sundberg EJ. 2016. Structural diversity in the type IV pili of multidrug-resistant *Acinetobacter*. J Biol Chem 291:22924–22935. doi:10.1074/jbc.M116.751099. 27634041 PMC5087714

[70] Ronish LA, Lillehoj E, Fields JK, Sundberg EJ, Piepenbrink KH. 2019. The structure of PilA from *Acinetobacter baumannii* AB5075 suggests a mechanism for functional specialization in *Acinetobacter* type IV pili. J Biol Chem 294:218–230. doi:10.1074/jbc.RA118.005814. 30413536 PMC6322890

[71] Meibom KL, Li XB, Nielsen AT, Wu CY, Roseman S, Schoolnik GK. 2004. The *Vibrio cholerae* chitin utilization program. Proc Natl Acad Sci U S A 101:2524–2529. doi:10.1073/pnas.0308707101. 14983042 PMC356983

[72] Metzger LC, Matthey N, Stoudmann C, Collas EJ, Blokesch M. 2019. Ecological implications of gene regulation by TfoX and TfoY among diverse *Vibrio* species. Environ Microbiol 21:2231–2247. doi:10.1111/1462-2920.14562. 30761714 PMC6618264

[73] Engler C, Kandzia R, Marillonnet S. 2008. A one pot, one step, precision cloning method with high throughput capability. PLoS One 3:e3647. doi:10.1371/journal.pone.0003647. 18985154 PMC2574415

[74] Bao Y, Lies DP, Fu H, Roberts GP. 1991. An improved Tn*7*-based system for the single-copy insertion of cloned genes into chromosomes of Gram-negative bacteria. Gene 109:167–168. doi:10.1016/0378-1119(91)90604-a. 1661697

[75] Keene ON. 1995. The log transformation is special. Stat Med 14:811–819. doi:10.1002/sim.4780140810. 7644861

[76] Lo Scrudato M, Blokesch M. 2012. The regulatory network of natural competence and transformation of *Vibrio cholerae*. PLoS Genet 8:e1002778. doi:10.1371/journal.pgen.1002778. 22737089 PMC3380833

[77] Metzger LC, Stutzmann S, Scrignari T, Van der Henst C, Matthey N, Blokesch M. 2016. Independent regulation of type VI secretion in *Vibrio cholerae* by TfoX and TfoY. Cell Rep 15:951–958. doi:10.1016/j.celrep.2016.03.092. 27117415 PMC4858559

[78] Craig L, Volkmann N, Arvai AS, Pique ME, Yeager M, Egelman EH, Tainer JA. 2006. Type IV pilus structure by cryo-electron microscopy and crystallography: implications for pilus assembly and functions. Mol Cell 23:651–662. doi:10.1016/j.molcel.2006.07.004. 16949362

[79] Matthey N, Drebes Dörr NC, Blokesch M. 2018. Long-read-based genome sequences of pandemic and environmental *Vibrio cholerae* strains. Microbiol Resour Announc 7:e01574-18. doi:10.1128/MRA.01574-18. 30574591 PMC6298558

[80] Traglia GM, Chua K, Centron D, Tolmasky ME, Ramirez MS. 2014. Whole-genome sequence analysis of the naturally competent *Acinetobacter baumannii* clinical isolate A118. Genome Biol Evol 6:2235–2239. doi:10.1093/gbe/evu176. 25164683 PMC4202317

[81] Simon R, Priefer U, Pühler A. 1983. A broad host range mobilization system for *in vivo* genetic engineering: transposon mutagenesis in Gram negative bacteria. Nat Biotechnol 1:784–791. doi:10.1038/nbt1183-784.

[82] Burrows LL. 2012. *Pseudomonas aeruginosa* twitching motility: type IV pili in action. Annu Rev Microbiol 66:493–520. doi:10.1146/annurev-micro-092611-150055. 22746331

[83] Heidelberg JF, Eisen JA, Nelson WC, Clayton RA, Gwinn ML, Dodson RJ, Haft DH, Hickey EK, Peterson JD, Umayam L, Gill SR, Nelson KE, Read TD, Tettelin H, Richardson D, Ermolaeva MD, Vamathevan J, Bass S, Qin H, Dragoi I, Sellers P, McDonald L, Utterback T, Fleishmann RD, Nierman WC, White O, Salzberg SL, Smith HO, Colwell RR, Mekalanos JJ, Venter JC, Fraser CM. 2000. DNA sequence of both chromosomes of the cholera pathogen *Vibrio cholerae*. Nature 406:477–483. doi:10.1038/35020000. 10952301 PMC8288016

[84] Barbe V, Vallenet D, Fonknechten N, Kreimeyer A, Oztas S, Labarre L, Cruveiller S, Robert C, Duprat S, Wincker P, Ornston LN, Weissenbach J, Marliere P, Cohen GN, Medigue C. 2004. Unique features revealed by the genome sequence of *Acinetobacter* sp. ADP1, a versatile and naturally transformation competent bacterium. Nucleic Acids Res 32:5766–5779. doi:10.1093/nar/gkh910. 15514110 PMC528795

[85] Winsor GL, Van Rossum T, Lo R, Khaira B, Whiteside MD, Hancock RE, Brinkman FS. 2009. *Pseudomonas* Genome Database: facilitating user-friendly, comprehensive comparisons of microbial genomes. Nucleic Acids Res 37:D483–D488. doi:10.1093/nar/gkn861. 18978025 PMC2686508

